# *Brucella*’s Emerging Threat: A Global Systematic Review and Meta‐Analysis Revealing Temporal, Geographic and Species‐Specific Patterns of Antimicrobial Resistance

**DOI:** 10.1155/vmi/8689240

**Published:** 2026-02-10

**Authors:** Gurkan Tut

**Affiliations:** ^1^ Department of Bacteriology, Animal and Plant Health Agency, Surrey, UK, vla.gov.uk

**Keywords:** antimicrobial resistance, *Brucella*, brucellosis, humans, meta-analysis, minimum inhibitory concentration testing, rifampicin, systematic review, trimethoprim-sulfamethoxazole

## Abstract

Antimicrobial resistance (AMR) can lead to treatment failure in human bacterial infections, resulting in increased morbidity and mortality. Brucellosis is a globally significant zoonotic infection caused by *Brucella* spp. bacteria, yet the frequency and extent of AMR in *Brucella* populations from humans are poorly characterised. This systematic review and meta‐analysis investigated AMR in populations of *Brucella* species responsible for the vast majority of brucellosis in humans (*B. melitensis*, *B. abortus*, *B. suis* and *B. canis*). The search and inclusion criteria identified studies which used testing methods of minimum inhibitory concentration (MIC: *E*‐test, broth and agar dilution), with the main (doxycycline, streptomycin and rifampicin) and alternative (trimethoprim‐sulfamethoxazole, gentamicin and ciprofloxacin) antibiotics utilised in the treatment of human brucellosis. Out of 704 studies identified via SCOPUS, 2 were non‐English, 401 were irrelevant, 230 were lacking key inclusion criteria and 20 had incomplete details and were excluded, leaving 51 for review; however, for *Z*‐tests and meta‐analysis, the absence of MIC_min/max_ data left 49 studies. The Newcastle–Ottawa Scale and the Grading of Recommendations, Assessment, Development and Evaluation systems were used to assess the risk of bias in the included studies. Two of the antibiotics considered (trimethoprim‐sulfamethoxazole and rifampicin) exhibited significant resistance. This resistance was reflected in 1‐sample *Z*‐tests, of which 39/228 (17.1%) produced an outcome of ‘non‐susceptible’ or ‘not proven to be susceptible’, and these predominantly belonged to trimethoprim‐sulfamethoxazole (15/34; 44.11%) and rifampicin (21/49; 42.85%). The type of meta‐analysis was generic inverse variance weighted average. For rifampicin and trimethoprim‐sulfamethoxazole, sensitivity analysis produced a MIC_50_ of 1.00 μg/mL (95% CI: 0.98, 1.01) and 0.50 μg/mL (95% CI: 0.49, 0.51), respectively. For rifampicin and trimethoprim‐sulfamethoxazole, AMR was linked to the years before 2010, non‐Asian continents and *Brucella* species when tested in mixed isolates. Therefore, more careful stewardship in the use of rifampicin and trimethoprim‐sulfamethoxazole antibiotics is necessary to prevent the development of AMR in *Brucella*.

## 1. Introduction

Brucellosis is a zoonotic disease which threatens human and animal health and adversely affects agricultural economies worldwide [1]. Brucellosis decreases livestock productivity, and in result undermines the livestock dependant populations food security and income [2]. *Brucella* spp. are fastidious, Gram‐negative coccobacilli, with several species within the genus being causative agents of brucellosis [1, 3]. *Brucella melitensis* (ovine and caprine livestock), *Brucella abortus* (bovine livestock), *Brucella suis* (swine and certain wildlife species) and *Brucella canis* (canines) are capable of causing human brucellosis, with *B. melitensis* the most frequent cause [4]. Other species of *Brucella* are pathogenic yet infrequently cause human infection (e.g., *Brucella ceti*), are considered nonpathogenic to humans (e.g., *Brucella ovis*), or are of, as yet, unascertained pathogenicity (e.g., *Brucella inopinata*) [4]. Infection of humans with *Brucella* primarily occurs by contact with infected livestock and their reproductive products, such as aborted foetuses and birth materials, and also with the consumption of unpasteurised dairy products [5].

Human brucellosis symptoms are not considered pathognomonic, with fever, chills, headaches, body aches, fatigue and joint and back pain frequently reported [6]. Both acute and chronic infections may impact all age groups and remain a public and animal health problem, especially in countries of the Middle East, Mediterranean Basin, Southern Europe, Central Asia, Latin America and North and East Africa [1, 3, 4]. Additionally, travel and migration of infected people and animals can result in the importation of *Brucella* cases to otherwise non‐endemic countries [4, 6]. Globally the WHO estimates half a million new cases of human brucellosis a year [7, 8]. Recently, an evidence‐based conservative estimate reported 2.1 million incidences annually worldwide [9]. *Brucella* was misclassified together with *Ochrobactrum*; however, recent genomic studies confirm despite their close relation, they are distinct genera [10]. In humans the standard treatment for brucellosis is based on administering oral doxycycline (DOX) and rifampicin (RIF) [11], as well as the use of DOX with streptomycin (STR) [12]. Trimethoprim‐sulfamethoxazole (SXT), ciprofloxacin (CIP) and gentamicin (GEN) are alternative antibiotic interventions used for combatting the high rate of relapse and treatment failure seen for brucellosis in humans [11–13]. RIF may be used together with SXT to treat brucellosis in children [14] and pregnant patients [15]. Additionally, the choice of antibiotic may be influenced by the clinical presentation, epidemiological factors and drug availability. In addition to this empirical treatment, the use of antimicrobial susceptibility testing can also aid in treatment decisions [11–13]. Antibiotic treatment options are not used for livestock, and strict prevention and control strategies are recommended [1]. For canine brucellosis antibiotic treatment is available with oxytetracycline, STR and enrofloxacin, but antibiotic treatment is strongly discouraged [16]. Livestock suffering from brucellosis are also able to acquire other acute infectious diseases such as tuberculosis. These infections are treated with the same antibiotics used against brucellosis, and consequently there is a potential for selection of resistance in *Brucella*, a pleiotropic effect [17–23].

Antimicrobial‐resistant *Brucella* isolates from humans have been described in several countries, including Egypt [24, 25], Qatar [26], Iran [27, 28], Kazakhstan [22], Inner Mongolia [19] and China [29]. Studies focused only on livestock are limited, but studies report antimicrobial resistance (AMR) in livestock exists in the Republic of Korea [30], Brazil [31], Mexico [32] and Iran [33]. Indiscriminate antimicrobial usage and the lack of appropriate antibiotic stewardship provide conditions where the emergence of AMR in *Brucella* is a risk [34]. Especially since the global burden of brucellosis in terms of disease incidence varies significantly within regions and countries, and aggregated data thus far has failed to capture the complexities of disease dynamics, developing the potential of at‐risk populations being overlooked, as brucellosis‐endemic countries lack strong health systems, which result in the passively acquired official data underestimating the real burden [35].

Especially in developing countries, brucellosis remains a significant zoonotic health concern where persistent transmission and relapse rates encumber control efforts. Relapses are prominently associated with AMR, which complicates treatment outcomes as well as contributes to increased morbidity. Occupational exposure between livestock carers, veterinarians and slaughterhouse workers increases the risk of disease transmission inherent to endemic regions [36]. On top of this, various socioeconomic, environmental and infrastructural factors, including but not limited to limited awareness and co‐operation in and between livestock handlers and veterinarians, as well as restricted access to accurate diagnostic tools, also play an important role in the ongoing persistence of brucellosis in these settings [37]. Tackling these multifaceted issues is fundamental for effective disease control and, most importantly, to mitigate the emergence and spread of AMR in endemic regions.

The nature of resistance in *Brucella* was assessed with a comprehensive systematic review and meta‐analysis on AMR in populations of *Brucella* which were tested with minimum inhibitory concentration (MIC) techniques, from species which cause brucellosis in humans (*B. melitensis*, *B. abortus*, *B. suis* and *B. canis*), for the main treatment options of DOX, STR and RIF, and the alternative treatment options of SXT, CIP and GEN. The nature of resistance was determined by comparing findings to CLSI breakpoints quoted in the most recent study on the topic [38].

## 2. Methods

This systematic review was conducted in accordance with PRISMA guidelines [39, 40].

### 2.1. Search Methods and Strategy

The search strategy aimed to identify all relevant published articles on AMR in *Brucella*. A comprehensive search strategy was performed across all publication years from 1951 to 2020, with the language restricted to English only. The search was conducted using the SCOPUS database. SCOPUS was chosen because of its extensive multidisciplinary coverage, which also includes indexing a broad range of journals, especially in biomedical, microbiological, veterinary and public health disciplines. Above all, the SCOPUS database contains content from many major bibliographic sources, such as Web of Science (> 99%) [41], Embase (> 99%) [42], Medline (> 99%) [42] and PubMed (> 85%) [43] via its platform. As a result, the use of SCOPUS ensures a maximal and comprehensive search scope.

The following search strategy was employed (search date 10th August 2020): (TITLE‐ABS‐KEY (antibiotic∗ OR antimicrobial∗ OR AMR) AND TITLE‐ABS‐KEY (resistance OR resistant OR susceptibility OR susceptible) AND TITLE‐ABS‐KEY (*Brucella* OR brucellosis) AND (LIMIT‐TO (LANGUAGE, “English”).

### 2.2. Selection Criteria

**FIGURE 1:**
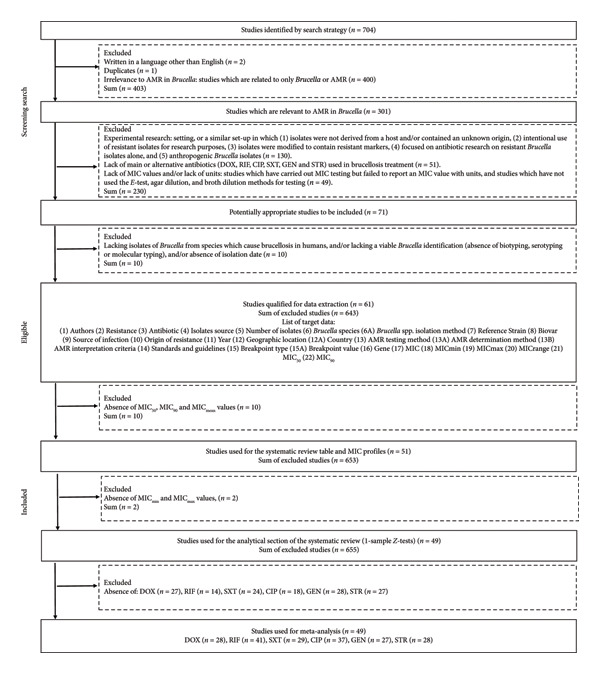
PRISMA flow chart of systematic search and selection of literature.

Studies reporting MIC testing of primary and alternative antibiotics against *Brucella* isolates from species responsible for human brucellosis were selected. Some studies were excluded because they exclusively focused on reporting MIC values for resistant *Brucella* isolates [44] and reported results of strains with mutations only [45]. Figure S1 displays the screening questions applied to pool relevant studies for data extraction. The screening process resulted in 61 studies qualifying for data extraction. The PRISMA flow chart (Figure 1) summarises the selection process for identifying studies that were eligible for the systematic review and meta‐analysis.

### 2.3. Data Extraction

The extracted data from the eligible 61 studies contained methodological quality, epidemiological factors, subject characteristics, *Brucella* species identification procedures and outcome data as defined in Figure 1. A full list and explanation of each data item are given in Table S1.

### 2.4. Data Calculation, Synthesis and Analysis

#### 2.4.1. Systematic Review

The systematic review aimed to collate (systematic review table) Table 1, summarise (MIC averages) and analytically investigate (1‐sample *Z*‐tests) the findings from each study. The extracted data (Table S2) was used to collate a systematic review table which provided information on key epidemiological and methodological variables alongside their reported MIC_50_ and MIC_90_.

**TABLE 1:** Summarised systematic review on AMR in *Brucella* ordered by publication year.

Study	*n*	Isolation year	Isolate source	*Brucella* species	Country	Continent	Isolation type	AMR testing method	DOX (*S* ≤ 4 μg/mL)		RIF (*S* ≤ 1)		CIP (*S* ≤ 1)		SXT (*S* ≤ 0.5)		GEN (*S* ≤ 4)		STR (*S* ≤ 8)	
									MIC_50_	MIC_90_	MIC_50_	MIC_90_	MIC_50_	MIC_90_	MIC_50_	MIC_90_	MIC_50_	MIC_90_	MIC_50_	MIC_90_
[46]	27	1970	Humans and animals	*B. suis* (10), *B. abortus* (7), *B. neotomae* (2), *B. canis* (4), *B. melitensis* (2) and *B. ovis* (2)	The United States and England	North America and Europe	Biotyping (outsourced)	Broth dilution	0.055	0.25	0.3	**1.25**	—	—	—	—	0.07	0.45	0.7	2.45
[47]	90	1972–1976	Canine	*B. canis*	Japan	Asia	Biotyping (outsourced)	Agar dilution	0.19	0.39	0.39	**1.56**	—	—	—	—	0.09	0.19	0.19	0.39
[48]	98	1981	Human	*B. melitensis*	Spain	Europe	Biotyping	Agar dilution	—	—	0.375	0.5	—	—	**1.23**	**4.5**	—	—	—	—
[49]	4–29	1980–1984	Human	*B. melitensis*	Spain	Europe	Biotyping and serotyping	Agar dilution	0.11^∗^	—	0.87^∗^	—	—	—	—	—	—	—	0.385^∗^	—
[50]	95	1980–1984	Human	*B. melitensis*	Spain	Europe	Biotyping	Agar dilution	0.12	0.25	**1**	**2**	0.25	0.5	0.12	0.25	—	—	0.5	1
[51]	8–15	1986	Human	*B. abortus* (4), *B. melitensis* (7), *B. suis* (2), *B. canis* (2)	The United States and Mexico	North America	Biotyping (outsourced)	Broth dilution	—	—	0.25	**1**	—	—	—	—	0.5	1	2	4
[52]	146	1989	Human	*B. melitensis* bv. 1 (94) and 3 (52)	Saudi Arabia	Asia	Biotyping	Broth dilution	—	—	0.25	**1**	0.25	0.5	**0.5**	**1**	0.25	0.25	—	—
[53]	114	1990	Human	*B. melitensis*	Saudi Arabia	Asia	Biotyping	Broth dilution	—	—	0.25	**1**	0.25	0.5	**0.5**	**1**	0.25	0.25	—	—
[54]	106	1987–1989	Human	*B. melitensis*	Saudi Arabia	Asia	Biotyping and serotyping	Agar dilution	—	—	**2**	**4**	**1**	**1**	**16**	**16**	—	—	2	4
[55]	139	1990–1991	Human	*B. melitensis* bv. 1 (91), 3 (48)	Saudi Arabia	Asia	Biotyping	Broth dilution	—	—	0.25	**1**	0.25	0.25	**0.5**	**1**	0.25	0.25	—	—
[56]	42	1991	Human	*B. melitensis*	Saudi Arabia	Asia	Biotyping	Agar dilution	—	—	—	—	0.12	0.5	0.25	**1**	0.25	0.5	—	—
[57]	86	1991	Human	*B. melitensis*	Israel	Asia	Biotyping and serotyping	Broth and agar dilution	—	—	**2.5**	**4**	0.4	0.8	**3.1**	**6.3**	—	—	1.25	3.1
[58]	105	1993	Human	*B. melitensis*	Saudi Arabia	Asia	Biotyping	Broth dilution	—	—	0.25	**1**	0.25	0.5	**0.5**	**1**	0.25	0.25	—	—
[59]	146	1993	Human	*B. melitensis* bv. 1 and 3	Saudi Arabia	Asia	Biotyping	Agar dilution	—	—	0.25	**1**	0.25	0.5	**0.5**	**1**	0.25	0.25	0.5	1
[60]	108	1993	Human	*B. melitensis*	Saudi Arabia	Asia	Biotyping	Agar dilution	—	—	—	—	0.25	0.5	—	—	—	—	—	—
[61]	126	1993	Human	*B. melitensis*	Saudi Arabia	Asia	Biotyping	Agar dilution	—	—	0.25	**1**	0.25	0.5	**0.5**	**1**	0.25	0.25	0.5	1
[62]	123	1994	Human	*B. melitensis* bv. 1, 2 and 3	Spain	Europe	Biotyping	Agar dilution	—	—	—	—	0.25	0.5	—	—	—	—	—	—
[63]	116	1995	Human	*B. melitensis* bv. 1 (86) and 3 (30)	Saudi Arabia	Asia	Biotyping	Broth dilution	—	—	0.25	**1**	0.25	0.5	**0.5**	**1**	0.25	0.5	—	—
[64]	41	2001–2002	Human	*B. melitensis* bv. 1 (2), 39 (3)	Turkey	Asia	Bio and serotyping	E‐test	0.047	0.064	0.5	0.75	0.125	0.25	0.094	0.38	—	—	—	—
[65]	42	2000–2003	Human	*B. melitensis* bv. 1 and 3, *B. abortus* bv. 3	Turkey	Asia	Biotyping	E‐test	0.032	0.064	0.75	**1**	0.094	0.19	**0.5**	**1.5**	—	—	—	—
[65]	37	2000–2003	Human	*B. melitensis* bv. 1 and 3	Turkey	Asia	Biotyping	E‐test	0.032	—	0.75		0.094		0.5	—	—	—	—	—
[65]	5	2000–2003	Human	*B. abortus* bv. 3	Turkey	Asia	Biotyping	E‐test	0.032	—	0.5	—	0.125	—	0.38	—	—	—	—	—
[66]	97	2004	Human, cow, goat and dog	*B. melitensis*, *B. abortus*, *B. suis* and *B. canis*	Mexico	North America	Biotyping	Agar dilution	0.25	0.5	**1**	**2**	0.5	0.5	**4**	**8**	—	—	1	4
[67]	44	1998–2003	Human	*B. melitensis*	Turkey	Asia	Biotyping	Agar dilution	0.25	0.5	—	—	0.5	**2**	—	—	—	—	—	—
[68]	74	1999–2005	Human, sheep and goat	*B. melitensis*	Crete, Cyprus and Syria	Asia	Biotyping	E‐test	—	—	0.5	**1**	0.19	0.5	—	—	0.19	2	1	2
[69]	50	1999–2003	Human	*B. melitensis* bv. 3	Turkey	Asia	Biotyping	Broth dilution	—	—	0.5	0.5	**1**	**1**	—	—	1	2	4	8
[70]	46	2002–2004	Human	B. melitensis bv. 3 (45) 1 (1)	Turkey	Asia	Biotyping	E‐test	—	—	0.5	**1**	0.125	0.25	—	—	—	—	0.25	0.5
[71]	60	2008	Human	*B. melitensis* bv. 3	Turkey	Asia	Bio and serotyping	E‐test	—	—	—	—	0.125	0.19	—	—	—	—	—	—
[72]	96	1991–2006	Human	*Brucella* spp.	Turkey	Asia	Biotyping	E‐test	—	—	**1**	**2**	0.19	0.38	0.032	0.125	—	—	1	2
[73]	77	2001–2006	Human	*B. melitensis* (76) and *B. abortus* (1)	Turkey	Asia	Bio‐and molecular typing	E‐test	0.047	0.125	0.75	**1.5**	0.19	0.38	0.047	0.094	0.25	0.5	0.5	1
[17]	56	2008–2009	Human	*B. melitensis*	Turkey	Asia	Biotyping	E‐test	0.047	0.064	1.5	2	—	—	0.064	0.125	—	—	1	1
[74]	25	2000–2001	Human	*B. melitensis* bv. 1	Peru	South America	Biotyping	E‐test	0.19	0.38	0.5	0.75	0.125	0.214	0.064	0.151	0.125	0.25	—	—
[74]	23	2005–2006	Human	*B. melitensis* bv. 1	Peru	South America	Biotyping	E‐test	0.38	0.38	0.38	0.5	0.125	0.25	0.032	0.094	0.125	0.226	—	—
[24]	355	1997–2007	Human	*B. melitensis*	Egypt	Africa	Bio‐ and molecular typing	E‐test	0.125	0.25	**2**	**4**	0.25	0.38	0.047	0.19	0.5	1	1.5	2
[30]	86	1998–2006	Cattle	*B. abortus*	South Korea	Asia	Bio‐ and molecular typing	Broth dilution	0.25	0.25	2	2	0.5	**1**	—	—	1	1	2	2
[75]	34	1999–2005	Human	*B. melitensis*	Turkey	Asia	Biotyping	Broth dilution	0.032	0.064	**1**	**2**	0.5	**1**	—	—	—	—	4	**8**
[76]	94	2002–2009	Human	*B. melitensis* bv. 3 (93), 1 (1)	Turkey	Asia	Bio‐ and molecular typing	E‐test	—	—	**1**	**1.5**	—	—	—	—	—	—	—	—
[20]	73	2009–2011	Human	*B. melitensis* bv. 3	Turkey	Asia	Bio‐ and molecular typing	E‐test	0.047	0.094	**1**	**2**	0.19	0.25	0.064	0.19	—	—	0.75	1
[29]	19	2010–2012	Human	*B. melitensis*	China	Asia	Biotyping and bacteria ID systems	E‐test	**16**	**32**	0.75	**2**	—	—	**8**	**16**	0.75	0.75	—	—
[77]	17	2011–2014	Human	*B. melitensis*	The United Kingdom	Europe	Biotyping	E‐test	—	—	—	**1**	—	0.25	—	—	—	0.25	—	1
[78]	41	2010‐2011	Human	B. melitensis bv. 3 2 and 1 (40) B. suis (1)	Malaysia	Asia	Bio and serotyping	E‐test	0.125	0.25	**1**	**1.5**	—	—	0.047	0.125	0.125	0.19	0.5	0.75
[26]	231	2005–2014	Human	*B. melitensis*	Qatar	Asia	Biotyping and bacteria ID systems	E‐test	0.125	0.25	**1**	**2**	0.25	0.5	0.064	0.25	0.25	0.5	0.5	2
[18]	50	2010–2012	Human	*B. melitensis*	Turkey	Asia	Biotyping and bacteria ID systems	E‐test	0.064	0.094	**1**	**1.5**	0.125	0.19	0.023	0.064	0.047	0.094	0.25	0.38
[31]	147	1977–2009	Cattle	*B. abortus* bv. 1, 2, 3, 4 and 6	Brazil	South America	Bio, sero and molecular typing	Agar dilution	0.5	0.5	**1**	**2**	0.5	0.5	—	—	1	1	2	2
[21]	48	2011–2013	Human	*B. melitensis*	Iran	Asia	Biotyping and bacteria ID systems	E‐test	0.047	0.1	0.38	0.75	0.25	0.38	0.016	0.064	—	—	0.625	1.5
[33]	38	2010–2015	Sheep (28) cattle (10)	*B. abortus* (2), *B. melitensis* (36)	Iran	Asia	Biotyping and bacteria ID systems	E‐test	0.125	—	0.064	**1**	0.125	—	0.064	0.125	0.125	—	1	—
[33]	30	2010–2015	Human	*B. abortus* (6), *B. melitensis* (24)	Iran	Asia	Biotyping and bacteria ID systems	E‐test	0.125	0.19	0.75	—	0.125	0.19	0.125	0.19	0.125	—	0.5	1
[79]	44	2014–2016	Human	*B. melitensis*	Turkey	Asia	Biotyping and bacteria ID systems	E‐test	0.24	1	0.032	0.5	0.5	0.5	—	—	—	—	0.5	0.5
[22]	329	2008–2014	Human	*B. melitensis*	Kazakhstan	Asia	Bio, sero and molecular typing	E‐test	0.047	0.094	**1.5**	**8**	—	—	—	—	1	3	0.75	3
[23]	57	2013–2014	Human	*B. melitensis*	Iran	Asia	Bio, sero and molecular typing	E‐test	0.47	0.47	**1**	**2**	0.25	0.38	0.25	**0.5**	0.38	0.75	1	1.5
[25]	66	2018	Human, Buffalo, cattle and sheep	*B. melitensis* bv. 3	Egypt	Africa	Bio‐serotyping and bacteria ID systems	Broth dilution	0.094	0.5	0.375	**1**	0.25	0.94	—	—	0.25	1	1.25	2.5
[80]	77	2000–2013	Human (children)	*B. melitensis* bv. 3	Turkey	Asia	Bio, sero and molecular typing	E‐test	0.032	0.047	**1**	**1**	—	—	0.023	0.032	0.38	0.5	—	—
[19]	85	2011–2015	Human	*B. melitensis*	Inner Mongolia (China)	Asia	Bio‐ and molecular typing	E‐test	0.125	0.25	0.5	**1**	0.5	0.5	**0.5**	**1**	0.5	1	—	—
[81]	54	2016–2018	Human	*B. melitensis* bv. 1	Iran	Asia	Bio and serotyping	E‐test	0.064	0.25	0.38	**1**	—	—	0.032	0.19	0.38	0.75	0.94	0.75
[82]	25	2018–2019	Human	*B. melitensis* bv. 1 and 3	Inner Mongolia (China)	Asia	Bio, sero, molecular typing and bacteria ID systems	Broth dilution	0.12	0.12	0.5	**1**	0.5	**1**	—	—	0.5	1	2	4

*Note:* MIC values are provided in concentrations of μg/ml. (—) study did not test the antibiotic and/or report the MIC value. The symbol (^∗^) indicates the author reported multiple mean values of MIC instead of MIC_50_, which were averaged for aesthetic viewing purposes. MIC_50_ and MIC_90_ values ≥ their CLSI breakpoints are in bold. A total of 51 studies contributed to this systematic review table. Bacteria identification systems: Bact/Alert 3D automatic blood culture system, VITEK II automatic bacteria analyser, BACTEC 9240 system, BacT/Alert and BACTEC FX.

Abbreviations: CIP, ciprofloxacin; DOX, doxycycline; GEN, gentamicin; RIF, rifampicin; STR, streptomycin; SXT, trimethoprim‐sulfamethoxazole.

##### 2.4.1.1. MIC Averages

MIC averages were produced by calculating the mean of MIC_50_, MIC_90_, MIC_min_ and MIC_max_ of the extracted data, using Minitab V.17 (Minitab LLC, Pennsylvania, USA). This calculation also included their standard errors (SEs), standard deviations and the total number of contributing studies. These profiles were used for observational comparisons with CLSI breakpoints.

##### 2.4.1.2. Interpretive Criteria

The interpretive criteria used were CLSI clinical breakpoints for *Brucella* MIC testing [38]; DOX susceptibility (*S*) ≤ 4 μg/mL, STR (*S* ≤ 8), GEN (*S* ≤ 4) and SXT (*S* ≤ 0.5). As recommended for RIF (*S* ≤ 1) and CIP (*S* ≤ 1), the CLSI breakpoints were extrapolated from the slow‐growing bacteria *Haemophilus* spp. [38].

##### 2.4.1.3. Occurrence of Resistance in *Brucella*

To determine if the reported MIC_50_ values from each study were statistically different from the CLSI breakpoint, 1‐sample *Z*‐tests were performed (MiniTab V.17). This analysis investigates the population of isolates from each study and evaluates how they sit in relation to the CLSI breakpoint. Consequently, sample size, MIC_mean_, range and standard deviation were required. Sample sizes determined how the estimated mean and standard deviation were calculated (see Table 3 from [83]). Executing 1‐sample *Z* tests provided the SE of the MIC_50_, which was used for subsequent meta‐analysis. The 1‐sample *Z*‐tests were used to infer the resistance status of the mean value, specifically whether it was statistically below (susceptible), not significantly different from (not proven to be susceptible), or above the CLSI breakpoint (resistant).

This statistical method tests the null hypothesis of if the MIC_50_ is equal to or different from (above or below) the CLSI susceptibility breakpoint, in turn providing a quantitative measure of resistance in *Brucella* populations at the individual study level. The advantage of this test is the facilitation for the calculation of the SE for the reported MIC_50_. The SE is acquired from the *Z* statistic, the reported MIC_50_, the sample size, the range and the standard deviation, and this is critical to be able to execute the subsequent single‐arm generic inverse variance weighted average (GIV WA) meta‐analytical test. The accurate estimation of the SE provides correct weighting of studies pooled in the single‐arm GIV WA meta‐analysis, in turn leading to more reliable pooled estimates. Therefore, the application of the 1‐sample *Z*‐test serves two key functions: (1) determining if each individual study’s reported MIC_50_ value is significantly different from the CLSI susceptibility breakpoint, which allows the measure of resistance in the *Brucella* populations and (2) generating the SE estimates necessary for being able to perform a single‐arm GIV WA meta‐analysis. Consequently, the 1‐sample *Z*‐test is an essential step in both resistance assessment on a study level and the ability to accurately synthesise data across multiple studies.

#### 2.4.2. Meta‐Analysis: Summarising MIC_50_ for Observational Comparisons With CLSI Breakpoints

Further investigation of the reported MIC_50_ values was performed with a single‐arm generic inverse variance (GIV) weighted average (WA) meta‐analysis on the MIC_50_ values utilising their SEs with the software ReviewManager 5.4 (Cochrane, Oxford, UK). This generated the GIV WA MIC_50_ and its 95% CIs for a direct observational comparison with the CLSI breakpoint. Studies which contained < 25 isolates were excluded from the meta‐analysis to limit the influence of small study effects [84].

Taking recommendations from [85], the GIV WA MIC_50_ and its upper and lower 95% confidence limits were visually compared to their CLSI breakpoints. The term ‘non‐susceptible’ was applied when the lower 95% confidence limit exceeded the CLSI breakpoint, ‘not proven to be susceptible’ was used when the 95% confidence limits spanned the CLSI breakpoint, and when the upper 95% confidence limit was below the CLSI breakpoint, the term ‘susceptible’ was used.

The studies and their relevant data (MIC_50_, SE, weight, MIC_50_ IV random 95% CIs) were subgrouped by four epidemiological factors: continent of isolation, isolate source (host species), *Brucella* sp. and year of isolation. Following this, studies and their relevant data were further subgrouped into their AMR testing methods and sample sizes.

#### 2.4.3. Meta‐Analysis: Investigating the Extent of AMR With Meta‐Analytical Tests

The second meta‐analysis addressed the extent of AMR in *Brucella* with statistical comparisons between the MIC_50_ and the CLSI breakpoints by investigating the statistical difference between the MIC_50_ and the CLSI breakpoint using the MIC_Diff_ (MIC_50_ − CLSI breakpoint). This permitted further investigation of the extent of resistance observed for each antibiotic and its subgroups with a *Z* test. *Z* tests identified whether GIV WA MIC_Diff_ values and their 95% confidence limits were significantly different from the CLSI breakpoint. MIC_Diff_ test results were categorised as either negative (susceptible), nonsignificant (not proven to be susceptible), or positive (resistant).

Studies and their relevant data were subgrouped (MIC_Diff_, SE, Weight, MIC_Diff_ IV Random 95% CIs), as described in Section 2.4.2. Forest plots were generated using ReviewManager (RevMan), which also tested for overall effect with a *Z* test, as described previously. The combination of both the *Z* and *p* values was used for interpreting the outcome [86].

Heterogeneity was evaluated with chi‐squared tests at a 5% level of significance, and inconsistency was measured with the *I* ^2^ statistic using ReviewManager. Type 1 errors were regulated through the use of Bonferroni correction [87], and Type 2 errors were regulated by adjusting significance to 0.01 when subgroups contained < 30 studies [88].

### 2.5. Bias and Quality Assessment

The Newcastle–Ottawa Scale (NOS) was modified to better fit the assessment of nonrandomised studies involved in AMR testing of *Brucella* and used to assess the risk of bias and methodological quality of studies which passed the screening process (see Figure S2) [89].

The Grading of Recommendations, Assessment, Development and Evaluations (GRADE) system was used to assess confidence in findings (cumulative estimate) of each study and the quality of each individual study included in the analysis [90] by evaluating the risk of bias, inconsistency, indirectness, imprecision and publication bias. Funnel plots were generated with ReviewManager; the asymmetry of data in the funnel plots was used to explore the potential reporting, selection and publication bias.

### 2.6. Sensitivity Analysis

For each antibiotic, sensitivity analysis was performed to produce a GIV WA MIC_50_ and MIC_Diff_ and their 95% CIs without the presence of inconsistency (*I* ^2^ statistic = 0%) and heterogeneity (Tau^2^ = 0.00; Chi^2^ ≤ 10.00), while employing a strategy to include the maximum number of possible studies [84]. This unravelled the potential impact of inconsistency and heterogeneity on found AMR, which was used to make a final decision on the nature of resistance.

## 3. Results

### 3.1. Paper Selection and Inclusion

The search strategy retrieved 704 articles in the SCOPUS database possibly related to AMR in *Brucella*. Following screening of titles, abstracts and full texts and removing duplicates, ineligible studies and those with insufficient data, 642 articles were excluded (see Figure 1). Table 1 presents 51 studies selected for the systematic review after excluding 10 studies that lacked MIC_50_ or MIC_90_ values. Initially, 61 studies met the inclusion criteria for data extraction, resulting in 289 data points summarised in Table S2. From these, an additional 10 studies were excluded from the systematic review because they did not report either MIC_50_ or MIC_90_ values. Furthermore, 2 studies were removed from the meta‐analysis due to missing MIC_min_ and MIC_max_ data, leaving 49 studies for the 1‐sample *Z*‐tests and meta‐analysis.

### 3.2. Systematic Review

#### 3.2.1. Narrative Summary

Data extracted from 61 studies resulted in 289 data points. Of these, 66.7% tested between 10 and 98 isolates, with 65.3% originating from Asia and the remaining 34.7% from Europe, the Americas and Africa. Out of agar dilution, broth dilution and *E*‐test, the latter was the most utilised (53.2%). *B. melitensis* (72.6%) and biovars 1 and 3 (92.5%) were the most frequently isolated species and reported biovars. The majority of isolates were obtained from humans (79.2%); the remaining 12.8% belong to animals only, and 8.0% contained a mixture of both animal and human isolates.

Author reports of encountering antimicrobial‐resistant *Brucella* were multicontinental at 30.4% (88/289). Resistance report rates: 65.0% (39/60) for RIF, 36.1% (17/47) for SXT, 26.4% (14/53) for CIP, 22.72% (10/44) for STR, 13.3% (6/45) for DOX and 5.0% (2/40) for GEN were found. Reports of RIF resistance occurred on all four continents, across several *Brucella* species and hosts. The mutated *rpoB* gene emerges as a possible contributor [26, 76]. Reports of SXT resistance have occurred in the last 2 decades and across all four continents (Asia, Europe, Africa and the Americas), with Asia at the forefront. Gaining an additional dihydrofolate reductase (DHPS) gene has been suggested as a contributor to SXT resistance [19].

Identification procedures of *Brucella* spp. extended with time, with all studies applying biotyping. The majority also employed the Rose Bengal test, Wright’s seroagglutination and the Coombs test [91], and then paired this with instruments (i.e., the Bact/Alert 3D automatic blood culture system). Only after 2010 did molecular typing (PCR) become common. The majority of studies considered that widespread use and misuse of antibiotics contributed to the presence of AMR in *Brucella*.

Table 1 summarises the studies included in the systematic review and provides details of the publication, the number of isolates tested (*n*), the year of isolation, the isolate source, the *Brucella* species, the country and continent from which the isolates originated, the isolation method and the AMR testing method used to determine the MIC_50_ and MIC_90_.

The studies in Table 1 are the source of studies used in all succeeding tables, this includes Tables 2–5.

**TABLE 2:** Mean MIC_50_ and MIC_90_ values of Brucella.

Antibiotic	Resistance^∗^	Mean MIC_50_	SE	MIC_50_ data set *n*	Mean MIC_90_	SE	MIC_90_ data set *n*	CLSI susceptibility breakpoints for *Brucella* (μg/mL) [38]
DOX	13.3% [29, 31, 49, 66, 67]	0.61	0.48	33	1.32	1.05	30	≤ 4
STR	22.7% [30, 31, 46, 49, 66, 69, 72, 75, 92]	1.13	0.16	32	2.16	0.33	32	≤ 8
RIF	65.0% [17–26, 29–33, 49, 50, 54, 57, 64–66, 68, 70, 72–76, 78, 80–82, 92–94]	0.74	0.07	48	1.58	0.18	46	≤ 1
SXT	36.1% [19, 23, 25, 29, 31–33, 65, 66, 68, 72, 81, 82, 95, 96]	1.11	0.50	42	1.95	0.70	42	≤ 0.5
GEN	5.0% [22, 31]	0.36	0.05	32	0.70	0.11	31	≤ 4
CIP	26.4% [20, 25, 30–33, 55, 58, 59, 63, 67, 72, 75, 97]	0.29	0.03	42	0.52	0.05	40	≤ 1

^∗^Percentage of reports of authors encountering resistant *Brucella* isolates. The ‘Study’ column in Table 1 contains details of relevant studies.

**TABLE 3:** Summarised meta‐analysis results of GIV WA of MIC_50_ and its 95% CIs on RIF and SXT for observational comparisons with CLSI breakpoints.

Subgroups	Factor	Data set *n*/subgroup *n*	MIC_50_ and 95% CIs (effect estimate)	Interpretation^A^
	RIF (studies *n* 41^∗^)		CLSI breakpoint ≤ 1 μg/ml^B^	

	Overall AMR [17–26, 30, 31, 33, 46–48, 50, 52–55, 58, 59, 61, 63–66, 68–70, 72–76, 78–82]	43	0.70 [0.59, 0.81]	Susceptible
Isolate source	Human isolates [17–24, 26, 48, 50, 52–55, 58, 59, 61, 63–65, 69, 70, 72–76, 78–82]	35	0.72 [0.60, 0.84]	Susceptible
	Mixed isolates [25, 30, 31, 33, 46, 47, 66, 68]	8	0.58 [0.37, 0.78]	Susceptible
Species	*B. melitensis* [17–26, 48, 50, 52–55, 58, 59, 61, 63, 64, 68–70, 74–76, 79–82]	32	0.68 [0.56, 0.81]	Susceptible
	*Brucella* spp. [30, 31, 33, 46, 47, 65, 66, 72, 73, 78]	11	0.74 [0.51, 0.98]	Susceptible
Continent	Asia [17–23, 26, 30, 33, 47, 52–55, 58, 59, 61, 63–65, 68–70, 72, 73, 75, 76, 78–82]	35	0.66 [0.54, 0.78]	Susceptible
	**Non-Asian** [24, 25, 31, 46, 48, 50, 66, 74]	8	0.85 [0.46, 1.25]	**Not proven to be susceptible**
Isolate isolation year	2000 and before [46–48, 50, 52–55, 58, 59, 61, 63]	12	0.34 [0.29, 0.38]	Susceptible
	**2001 to 2009** [17, 20, 22, 26, 64–66, 70, 73, 74, 76, 80]	13	0.89 [0.77, 1.02]	**Not proven to be susceptible**
	2010 and after [18, 19, 21, 23, 25, 33, 78, 79, 81, 82]	11	0.53 [0.34, 0.73]	Susceptible
	**Studies containing isolates before and after the year 2000** [24, 30, 31, 68, 69, 72, 75]	7	1.10 [0.50, 1.71]	**Not proven to be susceptible**

	SXT (studies *n* 29^∗^)		CLSI breakpoint ≤ 0.5 μg/ml^B^	

	**Overall AMR** [17–21, 23, 24, 26, 33, 48, 50, 52–56, 58, 59, 61, 63–66, 72–74, 78, 80, 81]	31	0.33 [0.28, 0.38]	Susceptible
Isolate source	Human isolates [17–21, 23, 24, 26, 48, 50, 52–56, 58, 59, 61, 63–65, 72–74, 78, 80, 81]	29	0.29 [0.24, 0.34]	Susceptible
	**Mixed isolates** [33, 66]	2	2.06 [0, 5.85]	**Not proven to be susceptible**
Species	*B. melitensis* [17–21, 23, 24, 26, 48, 50, 52–56, 58, 59, 61, 63, 64, 74, 80, 81]	24	0.38 [0.30, 0.46]	Susceptible
	*Brucella* spp. [33, 65, 66, 72, 73, 78]	7	0.36 [0.25, 0.48]	Susceptible
Continent	Asia [17–21, 23, 26, 33, 52–56, 58, 59, 61, 63–65, 72, 73, 78, 80, 81]	26	0.29 [0.24, 0.35]	Susceptible
	**Non-Asian** [24, 48, 50, 66, 74]	5	0.65 [0.50, 0.81]	**Not proven to be susceptible**
Isolate isolation year	**2000 and before** [48, 50, 52–56, 58, 59, 61, 63]	11	0.77 [0.63, 0.92]	**Non-susceptibility**
	2001 and after [17–21, 23, 26, 33, 64–66, 73, 74, 78, 80, 81]	18	0.10 [0.08, 0.12]	Susceptible
	Studies containing isolates before and after the year 2000 [24, 72]	2	0.05 [0.03, 0.06]	Susceptible

*Note:* Factors which are bold have been identified as containing GIV WA MIC_50_ and/or 95% CIs ≥ their CLSI breakpoint. The term **non-susceptibility** was restricted to all values (GIV MIC_50_ and its 95% CIs) exceeding the CLSI breakpoint. The term **not proven to be susceptible** terminology was used when more than one but less than three of the values (GIV MIC_50_ and its 95% CIs) exceeded the CLSI breakpoint, and when all values (GIV MIC_50_ and its 95% CIs) were below the CLSI breakpoint, the term **susceptible** was used for interpretation of AMR.

Abbreviation: CI, confidence interval.

^A^Interpretation according to [85] utilising the use of confidence intervals as a replacement for statistical significance tests.

^B^CLSI breakpoint [38].

^∗^The data set contained a greater *n* compared to studies because two studies reported multiple MIC values, arising from the testing of different *Brucella* species [65], or reported isolates due to certain epidemiological features such as isolate source [33]. The ‘Study’ column in Table 1 contains details of relevant studies.

**TABLE 4:** RIF and SXT’s summarised meta‐analysis results on the extent of AMR in *Brucella* defined by meta‐analytical tests.

Factor	*n*	MIC diff	95% CI lower	95% CI upper	Outcome of meta‐analysis			Interpretation	Outcome of heterogeneity tests	
					Test value	*p* value	*α*		*I* ^2^	*p* value
*RIF (studies 41)*										
**Overall AMR** [17–26, 30, 31, 33, 46–48, 50, 52–55, 58, 59, 61, 63–66, 68–70, 72–76, 78–82]	43	−0.30	−0.41	−0.19	*Z* = 5.46	(*p* < 0.00001)	0.00833	Susceptible	100%	(*p* < 0.00001)
**Epidemiological characteristics**										
Human isolates [17–24, 26, 48, 50, 52–55, 58, 59, 61, 63–65, 69, 70, 72–76, 78–82]	35	−0.28	−0.40	−0.16	*Z* = 4.58	(*p* < 0.00001)	0.00833	Susceptible	100%	(*p* < 0.00001)
Mixed isolates [25, 30, 31, 33, 46, 47, 66, 68]	8	−0.42	−0.63	−0.22	*Z* = 4.05	(*p* < 0.0001)	0.01	Susceptible	97%	(*p* < 0.00001)
**Comparison of isolate sources**	2				Chi^2^ = 1.33	(*p* = 0.25)	0.05	No difference	25.1%	—
*B. melitensis* [17–26, 48, 50, 52–55, 58, 59, 61, 63, 64, 68–70, 74–76, 79–82]	32	−0.32	−0.44	−0.19	*Z* = 5.00	(*p* < 0.00001)	0.00833	Susceptible	100%	(*p* < 0.00001)
*Brucella* spp. [30, 31, 33, 46, 47, 65, 66, 72, 73, 78]	11	−0.26	−0.49	−0.02	*Z* = 2.15	(*p* = 0.03)	0.01	**Nonsignificant**	97%	(*p* < 0.00001)
**Comparison of *Brucella* species**	2				Chi^2^ = 0.21	(*p* = 0.65)	0.05	No difference	0%	—
Asia [17–23, 26, 30, 33, 47, 52–55, 58, 59, 61, 63–65, 68–70, 72, 73, 75, 76, 78–82]	35	−0.34	−0.46	−0.22	*Z* = 5.69	(*p* < 0.00001)	0.00833	Susceptible	100%	(*p* < 0.00001)
Non‐Asian [24, 25, 31, 46, 48, 50, 66, 74]	8	−0.15	−0.54	0.25	*Z* = 0.74	(*p* = 0.46)	0.01	**Nonsignificant**	99%	(*p* < 0.00001)
**Comparison of geography**	2				Chi^2^ = 0.83	(*p* = 0.36)	0.05	No difference	0%	
Isolation year of ≤ 2000 [46–48, 50, 52–55, 58, 59, 61, 63]	12	−0.66	−0.71	−0.62	*Z* = 27.18	(*p* < 0.00001)	0.01	Susceptible	96%	(*p* < 0.00001)
Contains isolation years of 2001–2009 [17, 20, 22, 26, 64–66, 70, 73, 74, 76, 80]	13	−0.11	−0.23	0.02	*Z* = 1.62	(*p* = 0.11)	0.01	**Nonsignificant**	98%	(*p* < 0.00001)
Isolation year of ≥ 2010 [18, 19, 21, 23, 25, 33, 78, 79, 81, 82]	11	−0.47	−0.66	−0.27	*Z* = 4.70	(*p* < 0.00001)	0.01	Susceptible	98%	(*p* < 0.00001)
Overlapping isolation years of ≤ 2000 and ≥ 2000 [24, 30, 31, 68, 69, 72, 75]	7	0.10	−0.50	0.71	*Z* = 0.34	(*p* = 0.74)	0.01	**Nonsignificant**	99%	(*p* < 0.00001)
**Comparing time of isolation**	4				Chi^2^ = 70.17	(*p* < 0.00001)	0.05	**Difference**	95.7%	—

*SXT (studies 29)*										
**Overall AMR** [17–21, 23, 24, 26, 33, 48, 50, 52–56, 58, 59, 61, 63–66, 72–74, 78, 80, 81]	31	−0.17	−0.22	−0.12	*Z* = 6.91	(*p* < 0.00001)	0.00833	Susceptible	100%	(*p* < 0.00001)
**Epidemiological characteristics**										
Human isolates [17–21, 23, 24, 26, 48, 50, 52–56, 58, 59, 61, 63–65, 72–74, 78, 80, 81]	29	−0.21	−0.26	−0.16	*Z* = 8.27	(*p* < 0.00001)	0.01	Susceptible	100%	(*p* < 0.00001)
Mixed isolates [33, 66]	2	1.56	−2.24	5.35	*Z* = 0.80	(*p* = 0.42)	0.01	**Nonsignificant**	100%	(*p* < 0.00001)
**Comparison of isolate sources**	2				Chi^2^ = 0.83	(*p* = 0.36)	0.05	No difference	0%	—
*B. melitensis* [17–21, 23, 24, 26, 48, 50, 52–56, 58, 59, 61, 63, 64, 74, 80, 81]	24	−0.12	−0.20	−0.04	*Z* = 2.84	(*p* = 0.005)	0.01	Susceptible	100%	(*p* < 0.00001)
*Brucella* spp. [33, 65, 66, 72, 73, 78]	7	−0.14	−0.25	−0.02	*Z* = 2.30	(*p* = 0.02)	0.01	**Nonsignificant**	98%	(*p* < 0.00001)
**Comparison of *Brucella* species**	2				Chi^2^ = 0.07	(*p* = 0.80)	0.05	No difference	0%	—
Asia [17–21, 23, 26, 33, 52–56, 58, 59, 61, 63–65, 72, 73, 78, 80, 81]	26	−0.21	−0.26	−0.15	*Z* = 7.55	(*p* < 0.00001)	0.01	Susceptible	100%	(*p* < 0.00001)
Non‐Asian [24, 48, 50, 66, 74]	5	0.15	−0.00	0.31]	*Z* = 1.92	(*p* = 0.05)	0.01	**Nonsignificant**	99%	(*p* < 0.00001)
**Comparison of geography**	2				Chi^2^ = 18.41	(*p* < 0.0001)	0.05	**Difference**	94.6%	—
Isolation year of ≤ 2000 [48, 50, 52–56, 58, 59, 61, 63]	11	0.27	0.13	0.42	*Z* = 3.72	(*p* = 0.0002)	0.01	**Non-susceptible**	100%	(*p* < 0.00001)
Isolation year of > 2000 [17–21, 23, 26, 33, 64–66, 73, 74, 78, 80, 81]	18	−0.40	−0.42	−0.38	*Z* = 35.20	(*p* < 0.00001)	0.01	Susceptible	98%	(*p* < 0.00001)
Overlapping isolation years of ≤ 2000 and ≥ 2000 [24, 72]	2	−0.45	−0.47	−0.44	*Z* = 68.85	(*p* < 0.00001)	0.01	Susceptible	0%	(*p* = 0.98)
**Comparing time of isolation**	3				Chi^2^ = 111.28	(*p* < 0.00001)	0.05	**Difference**	98.2%	—

*Note:* Factors which are bold have been identified as containing GIV WA MIC_50_ and/or 95% CIs ≥ their CLSI breakpoint. The *I* ^2^ and *p* value were calculated for the heterogeneity test by ReviewManager. The ‘Study’ column in Table 1 contains details of relevant studies.

^a^MIC_diff_ calculated by MIC_50_ − CLSI breakpoint, CI = confidence interval, *α* = the alpha level used to determine significance, *I* ^2^ = inconsistency value.

**TABLE 5:** Sensitivity analysed meta‐analysis results on AMR in *Brucella* defined by GIV WA MIC_50_ and MIC_Diff_ in the absence of heterogeneity.

Antibiotic	Studies	MIC_50_ and 95% CIs (effect estimate)	Interpretation^A^	MIC50 − CLSI breakpoint = MIC diff	Outcome of meta‐analysis	*α* (*p* value significance threshold)	Interpretation^B^	Outcome of heterogeneity tests
DOX	5 [19, 24, 26, 33, 78]	0.13 [0.12, 0.13]	Susceptible	−3.87 [−3.88, −3.87]	Test for overall effect: *Z* = 1550.41 (*p* < 0.00001)	0.01	Susceptible	Heterogeneity: Tau^2^ = 0.00; Chi^2^ = 0.00, d*f* = 5 (*p* = 1.00); *I* ^2^ = 0%
STR	8 [26, 33, 50, 59, 61, 73, 78, 79]	0.50 [0.48, 0.52]	Susceptible	−7.50 [−7.52, −7.48]	Test for overall effect: *Z* = 913.56 (*p* < 0.00001)	0.01	Susceptible	Heterogeneity: Tau^2^ = 0.00; Chi^2^ = 0.00, d*f* = 7 (*p* = 1.00); *I* ^2^ = 0%
RIF	12 [18, 20, 23, 26, 31, 33, 50, 66, 75, 76, 78, 80]	1.00 [0.98, 1.01]	**Not proven to be susceptible**	−0.00 [−0.02, 0.01]	Test for overall effect: *Z* = 0.28 (*p* = 0.78)	0.01	**Nonsignificant**	Heterogeneity: Tau^2^ = 0.00; Chi^2^ = 8.45, d*f* = 11 (*p* = 0.67); *I* ^2^ = 0%
SXT	9 [19, 52, 53, 55, 58, 59, 61, 63, 65]	0.50 [0.49, 0.51]	**Not proven to be susceptible**	0.00 [−0.01, 0.01]	Test for overall effect: *Z* = 0.00 (*p* = 1.00)	0.01	**Nonsignificant**	Heterogeneity: Tau^2^ = 0.00; Chi^2^ = 0.00, d*f* = 9 (*p* = 1.00); *I* ^2^ = 0%
GEN	11 [25, 26, 52, 53, 55, 56, 58, 59, 61, 63, 73]	0.25 [0.25, 0.25]	Susceptible	−3.75 [−3.75, −3.75]	Test for overall effect: *Z* = 1946.86 (*p* < 0.00001)	0.01	Susceptible	Heterogeneity: Tau^2^ = 0.00; Chi^2^ = 0.00, d*f* = 10 (*p* = 1.00); *I* ^2^ = 0%
CIP	14 [21, 23–26, 52, 53, 55, 58–63]	0.25 [0.25, 0.25]	Susceptible	−0.75 [−0.75, −0.75]	Test for overall effect: *Z* = 393.04 (*p* < 0.00001)	0.01	Susceptible	Heterogeneity: Tau^2^ = 0.00; Chi^2^ = 0.00, d*f* = 13 (*p* = 1.00); *I* ^2^ = 0%

*Note:* Bold values represent GIV WA MIC50 and/or 95% CIs ≥ of their CLSI breakpoint.

^A^Interpretation according to [85] utilising the use of confidence intervals as a replacement for statistical significance tests.

^B^Interpretation according to the outcome of meta‐analytical tests. The ‘Study’ column in Table 1 contains details of relevant studies.

#### 3.2.2. MIC Averages

Table 2 displays the mean MIC_50_ and MIC_90_, and Table S3 displays the mean MIC_max_ and MIC_min_ of the six antibiotics. As shown in Table 2, SXT contains a mean MIC_50_ value that is more than double the CLSI (*S*) breakpoint. Furthermore, the mean MIC_90_ value of RIF and SXT also exceeds the CLSI (*S*) breakpoint.

#### 3.2.3. Occurrence of Resistance in *Brucella*

From the 228 1‐sample *Z*‐tests performed, the comparison of the MIC_50_ to the CLSI breakpoints produced 39 outcomes of nonsignificance (not proven to be susceptible) or indicating non‐susceptibility in the population. This equates to an overall antibiotic non‐susceptible or susceptibility not proven occurrence rate of 17.1%. These test outcomes were predominantly observed for RIF (Figure S3) and SXT (Figure S4); for SXT this was at 44.11% (15/34 data points), while for RIF an occurrence rate of 42.85% (21/49) was found. For CIP the occurrence rate was 4.87% (2/41), for DOX 2.7% (1/37) and for GEN and STR 0% (0/32 and 0/35, respectively) was found.

### 3.3. Meta‐Analysis

**FIGURE 2:**
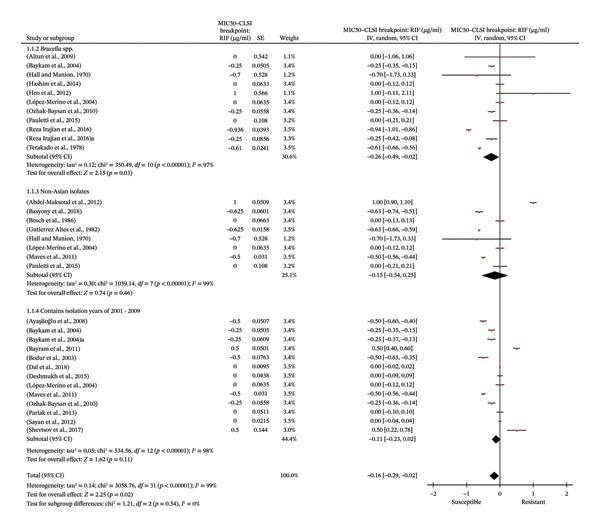
Forest plot of the meta‐analysis on the extent of RIF resistance of *Brucella* in certain epidemiological factors. In Figure 2 individual study results are represented with a red dot which is accompanied by their 95% CI bars. The black line in the middle represents the CLSI breakpoint, and the black diamond is the overall effect.

**FIGURE 3:**
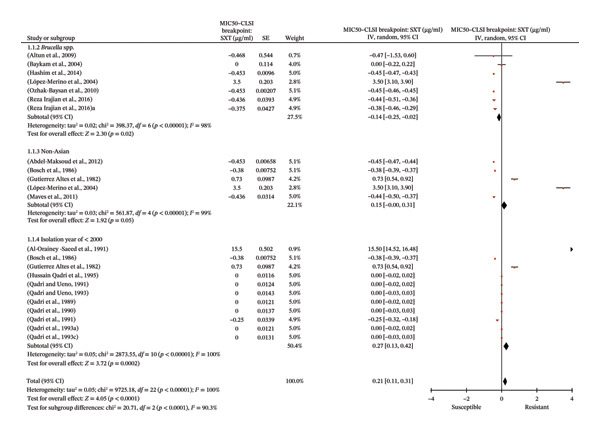
Forest plot of the meta‐analysis on the extent of SXT resistance of *Brucella* in certain epidemiological factors. In Figure 3 individual study results are represented with a red dot which is accompanied by their 95% CI bars. The black line in the middle represents the CLSI breakpoint, and the black diamond is the overall effect.

Forty‐nine studies qualified for meta‐analysis (see Section 3.1 and Figure 1). The pooled GIV WA of MIC_50_ and its 95% CIs for RIF and SXT are shown in Table 3. Table 4 shows reported antimicrobial susceptibility and/or resistance in *Brucella* in comparison to the CLSI breakpoints. Non‐susceptible and not proven to be susceptible *Brucella* were found for RIF (Figure 2 and Table 4) and SXT (Figure 3 and Table 4). Intriguingly, in RIF and SXT, non‐susceptibility and nonsignificant *Brucella* subgroups were associated with certain epidemiological factors. For both types of meta‐analysis and the antibiotics era, in which before the year 2000 for SXT and 2001–2009 for RIF, as well as non‐Asian isolate subgroups, were associated with non‐susceptibility and/or nonsignificance.

#### 3.3.1. Meta‐Analysis: Summarising MIC_50_ for Observational Comparisons With CLSI Breakpoints

*Brucella* bacteria were susceptible to DOX, STR, GEN and CIP under all factors (GIV WA MIC_50_ and 95% CIs below their respective CLSI breakpoints; see Table S4). For RIF, 3 of 17 tested factors, and for SXT, 5 of 16 factors, contained GIV WA MIC_50_ and 95% CIs ≥ the CLSI breakpoint of 1 μg/mL and 0.5 μg/mL, respectively (see Tables 3 and S4).

Table 3 summarises the meta‐analysis results on the GIV WA of MIC_50_ and its 95% CIs for RIF and SXT. Subgroups are represented by the factor column and are followed by the number of contributing data points and then by the GIV WA of MIC_50_ and its 95% CIs. Summarised MIC_50_ and the 95% CIs were compared to the CLSI breakpoint to define the susceptibility profile in each antibiotic × factor to visually determine their AMR profile.

#### 3.3.2. Meta‐Analysis: Investigating the Extent of AMR With Meta‐Analytical Tests

Forest plots (Figures 2 and 3) highlight the key epidemiological subgroups of non‐susceptible and/or not proven to be susceptible for RIF and SXT, respectively. As shown in Figure 2, the forest plot demonstrates the overall effect (black diamond). The displayed subgroups of *Brucella* spp. (*p* = 0.03), non‐Asian isolates (*p* = 0.46), and studies containing isolates between the years of 2001 and 2009 (*p* = 0.11) were all nonsignificantly different from the CLSI breakpoint of RIF (*S* ≤ 1 µg/ml). For SXT in Figure 3, this was *Brucella* spp. (*p* = 0.02) and non‐Asian isolates (*p* = 0.05), while the isolation year of ≤ 2000 was non‐susceptible (*p* = 0.0002), since the significant difference was due to being above the CLSI breakpoint of SXT (*S* ≤ 0.5 µg/ml). The Bonferroni‐corrected α threshold was used for interpretations.

Table 4 summarises the meta‐analysis results for RIF and SXT. The MIC_Diff_ represents the difference from the designated CLSI breakpoint. *Brucella* bacteria were susceptible to DOX, STR, GEN and CIP under all factors (see Table S4). As shown in Table 4 for RIF, five factors were found to be nonsignificant (*p* > 0.01) (not proven to be susceptible); for SXT, this was four factors. SXT also contained another two factors which were non‐susceptible *p* < 0.01 (see Table 4).

For all six antibiotics, chi‐squared tests indicated the presence of considerable heterogeneity (*p* < 0.01; *I* ^2^%: > 75%). Consistent with the findings of the systematic review, AMR in *Brucella* was found with meta‐analysis for RIF and SXT.

As demonstrated in Table 4, exploration of differences in GIV WA MIC_Diff_ of subgroups with chi‐squared tests identified several differences. Interestingly, the AMR testing method resulted in a difference in MIC_Diff_ for all tested antibiotics (*p* < 0.05). For instance, in the RIF E‐test method was nonsignificantly different from the CLSI breakpoint (*p* = 0.03), while for SXT this was broth dilution (*p* = 1.00), and agar dilution was non‐susceptible (*p* < 0.00001). Meta‐analysis was also performed with the use of MIC_mean_ instead of MIC_50_ (see Tables S5 and S6), which produced consistent findings to those described above; however, performing the meta‐analysis on MIC_mean_ pushed the findings on non‐susceptibility and/or nonsignificance (not proven to be susceptible) into overall AMR in *Brucella* for RIF and SXT and increased this in certain epidemiological factors.

#### 3.3.3. Quality Assessment

Table S7 summarises the quality evaluation for each primary outcome in GRADE. As all studies were nonrandomised, this played a key role in evaluation. The majority of studies (45/61) fulfilled 4 out of 5 quality domains and scored an overall GRADE of moderate with a strong recommendation.

Summaries of the methodological quality with the modified NOS scale for the included studies are available in Table S8. From the three quality domains (selection, comparability and outcome), the majority of studies scored similarly and were awarded between 6 and 7 stars out of 9.

#### 3.3.4. Sensitivity Analysis, Assessing Heterogeneity and Publication Bias

As expected from nonrandomised studies, for each of the six antibiotics, significant heterogeneity existed between studies (*p* < 0.01). The nonrandomised nature of studies and variation in the MIC_Diff_ values between studies contributed considerably to this outcome. As anticipated, differences in the GIV WA MIC_Diff_ and the 95% CIs due to epidemiological factors and/or meta‐biases failed to resolve the presence of considerable heterogeneity.

Removing inconsistency (*I* ^2^ statistic = 0%) and heterogeneity (Tau^2^ = 0.00; Chi^2^ < 10.00) while also preserving the maximum number of possible studies resulted in RIF (*p* = 0.78) and SXT (*p* = 1.00) becoming nonsignificant, in which the susceptibility status could not be proven when compared to their CLSI (*S*) breakpoints (Table 5). While DOX, STR, GEN and CIP remained susceptible. The removal of inconsistency (*I* ^2^ = 0%) and heterogeneity chi‐squared test (*p* ≥ 0.67) was successful for all antibiotics. Findings from the sensitivity analysis enabled a final decision on the nature of resistance of *Brucella* to each antibiotic (Table 5). Findings from the funnel plots suggest the possibility of publication and other biases since they exhibited asymmetry. For funnel plots of RIF and SXT, refer to Figures S5 and S6, respectively.

## 4. Discussion

This comprehensive systematic review and meta‐analysis is the first to quantify and examine the global extent of AMR in *Brucella*, alongside synthesising available microbiological MIC_50_ data on *Brucella* populations from species responsible for human brucellosis.

The results suggest *Brucella* populations of *B. abortus*, *B. melitensis*, *B. suis* and *B. canis* remain susceptible to DOX, STR, GEN and CIP. However, evidence indicates some populations are either non‐susceptible or lack clear susceptibility to RIF and SXT. Notably, the discovery of resistance to SXT is significant, because this antibiotic is recommended by WHO and is key for treating children, infants [98, 99] and pregnant women [100]. Interestingly, for other infectious disease‐causing bacteria, trimethoprim and sulfamethoxazole were combined to combat AMR in these two antibiotics [101].

For RIF, reports of *Brucella* resistance from studies had a global rate of 65%, the highest of all six antibiotics, and this translated into the mean MIC_90_ of 1.58 μg/mL (see Table 2), suggesting 10% of the *Brucella* population is non‐susceptible to RIF. Meta‐analysis produced a GIV WA MIC_50_ of 1.00 μg/mL (95% CI: 0.98, 1.01 μg/mL) without heterogeneity, and consequently a nonsignificant difference from the CLSI breakpoint of 1.00 μg/mL was found post sensitivity analysis (not proven to be susceptible status). Currently, there are no studies which investigate AMR in *Brucella* at a global scale; as a result, no studies for direct comparison are available, and despite this, 37 studies across the globe agree with findings on RIF and report MIC_50_ and/or MIC_90_ values ≥ 1.00 μg/mL (see Table 1). The widespread and intensive use of RIF as a first‐line agent against tuberculosis in brucellosis‐endemic regions has been proposed as a possible driver of RIF resistance in *Brucella* [17–23]. In some studies, polymorphisms in the *rpoB* gene have also been reported in RIF‐resistant *Brucella* isolates [26, 76].

For SXT, reports of *Brucella* resistance from studies were relatively frequent (36.1%) and were made evident by the mean MIC_50_ (1.11 μg/mL) and MIC_90_ (1.95 μg/mL) values (see Table 2), suggesting the mean *Brucella* population is non‐susceptible to SXT. In addition to this, the GIV WA MIC_50_ and 95% CIs (0.50; 0.49, 0.51 μg/mL) spanned the CLSI breakpoint of ≤ 0.5 μg/mL [38, 102], following sensitivity analysis. SXT resistance in *Brucella* species was highlighted decades ago by several publications [103, 104]. Collectively, worldwide, 16 studies will agree with these findings (see Table 1), and taking this evidence into account, SXT should not be considered, especially for monotherapy. Another study investigated the treatment of brucellosis in pregnant women and found the ceftriaxone/RIF combination as an effective alternative [105]. Potential alternatives such as macrolides are already met with resistance [92, 106].

The origin of AMR to RIF and SXT is reported to involve intricate genetic and clinical factors. Studies reported RIF resistance was directed by mutations in the *rpoB* gene, which encodes the β‐subunit of RNA polymerase, in turn reducing RIF binding of the target site and conferring resistance [26, 76, 93, 94]. RIF resistance impairs efficacy and leads to treatment failure, as well as increasing transmission rates, especially in tuberculosis‐prevalent regions where misuse may occur [18–23]. Some studies suggested efflux pumps expelling the antibiotic from the cell [19, 31]. The common theme across the majority for substantiating RIF resistance entails widespread/intensive/inappropriate misuse [17, 75, 81, 82], highlighting the need for better antimicrobial stewardship. Likewise, SXT resistances are reported to involve several mechanisms. Resistance can be obtained from alterations in genes *folA* and *folP* [93] encoding an altered enzyme for dihydrofolate reductase (DHFR) or deoxyhypusine synthase (DHPS) [19], alongside mutations which reduce drug binding affinity [31]. The widespread/intensive/inappropriate misuse of SXT also plays a major role in resistance [20, 23, 81, 82, 107]. From a clinical standpoint the misuse of SXT can foster the return of resistant strains and hinder treatment outcome, especially for children, infants [98, 99] and pregnant women [100]. Interestingly, earlier studies on RIF and SXT resistance identified prior use of fluoroquinolones, especially CIP, as promoting cross‐resistance [52, 53, 55, 58, 59, 61]. These findings on reports of the origin of resistance underline the significance of responsible antibiotic stewardship to preserve the efficacy of both these antibiotics.

The stratification analysis focused on epidemiological and meta‐bias characteristics. GIV WA of MIC_Diff_ for all six antibiotics was impacted by AMR testing methods (*p* < 0.05), introducing the possibility of methods having different sensitivities. But the applied methods are fixed into specific time periods, and as a result this invalidated this finding. For instance, *E*‐test was especially popular after 2010, and agar dilution prior to the year 2000. Such findings remind us to consider methodologies for systematic reviewing and meta‐analysis when utilising the results of different studies; this was also another reason for the focus on MIC methods.

From the tested epidemiological subgroups, an emerging pattern in which the *Brucella* spp. were not proven to be susceptible (nonsignificant) for both RIF and SXT was found. This subgroup is key in this study, since it represents the populations of *Brucella* from species which cause brucellosis in humans found in animal husbandry. Animal husbandry is a major player in the life cycle of brucellosis and involves raising animals and/or the production of animal products, which require the use of antibiotics to manage both animal and human health [1, 3, 4].

Interestingly, when GIV WA MIC_Diff_ and 95% CIs differences were found in continents between Asia and non‐Asia (majority originating from Europe), Asia contained reduced MIC_Diff_ (SXT *p* < 0.0001, and DOX *p* = 0.0006). Also, another branch of AMR copulated into non‐Asian isolates for SXT (0.65; 95% CIs 0.50, 0.81 μg/ml) and RIF (0.85; 95% CIs 0.46, 1.25 μg/mL). The role of different socioeconomic statuses between continents and countries alongside their varying medical and public health degrees possibly contributes to this outcome. For instance, differences in resistance may be caused by (1) ease of administration and lower cost of DOX plus RIF [108]; (2) popularity [109]; (3) nonrecommended regime usages of RIF combined with SXT [110–112]; and (4) lower capability in medical assistance and personnel for parenteral administration of STR and GEN [113, 114]. Such circumstances may have fuelled AMR in *Brucella* in specific eras.

Endemic areas such as the Middle East and North Africa regions are especially challenged in providing effective treatment. Factors including limited access to advanced diagnostic facilities, varying application of control and vaccination programmes in livestock, and widespread misuse of antibiotics are contributors to the emergence of AMR and relapses in treatment. In these endemic areas optimal therapeutic outcome is impeded by socio‐economic, cultural and infrastructural limitations [115]. Our findings of resistance in RIF and SXT align well with challenges faced in endemic areas and bring forward the microbiological evidence. Overcoming the therapeutic challenges demands a multifaceted approach in improving surveillance systems, appropriate stewardship of antibiotic use, and the implementation of One Health strategies which cover animal and human health sectors [115].

Changes in GIV WA MIC_Diff_ and 95% CIs values observed over time for RIF, SXT, STR, GEN and CIP (*p* < 0.05), were possibly affected by standard surveillance and treatment programmes of brucellosis [116]. Initially, SXT was possibly exploited to manage human and animal health, which resulted in the GIV WA MIC_50_ and 95% CIs becoming almost double the breakpoint value (0.77 μg/mL: 0.63, 0.92) before the year 2000. This caused the publication of critical studies [103, 104] which brought attention to the issue and hindered SXT resistance in the following decades. Yet the demand for antibiotics for managing human and animal health remained. Consequently, RIF was possibly exploited to fill in the gap, which caused the GIV WA MIC_50_ and its 95% CIs to reach an extent of meeting and/or exceeding the CLSI breakpoint (0.89 μg/mL: 0.77, 1.02), especially between the isolation years of 2001 and 2009. The surge in RIF resistance of *Brucella* was met by several studies [108, 109, 113], alongside the rollout of the European Centre for Disease Prevention and Control deciding in 2008 to establish the European Antibiotic Awareness Day. The combination of these measures limited further RIF resistance of *Brucella*.

Relying on a single database may pose a limitation; however, the comprehensive coverage provided by SCOPUS mitigates potential selection bias by covering many key bibliographic sources [41–43]. Importantly, the broad search strategy, combined with focussing on the SCOPUS database, ensured both efficiency and consistency. Considerable heterogeneity (75%–100%) was expected due to the use of nonrandomised studies and the all‐inclusive approach of combining a large pool of studies from a diverse number of settings [84]. Sensitivity analysis demonstrated the influence of heterogeneity on the summarised effect estimates (MIC_50_/MIC_Diff_ and 95% CIs) contributes insignificantly to the nature of the reported AMR in *Brucella* for DOX, STR, GEN and CIP, while the contrary was true for RIF and SXT.

Previous reports identified resistance issues in *Brucella,* yet this systematic review and meta‐analysis uniquely highlighted the evolution of AMR across different regions, host species and eras. Most importantly, identifying geographic disparities and temporal shifts in resistance patterns underlines the dynamic nature of *Brucella* AMR, as well as the necessity of ongoing global surveillance and stewardship. Moreover, these findings provide a mechanistic and clinical context for grasping the emerging resistance trends, and as a result, contribute significant new insights to the field.

This study’s contributions to the investigation of AMR in *Brucella* and identification of RIF and SXT resistance with a microbiological basis, combined with the failure of treatment from relevant systematic reviews and meta‐analyses [11–13, 117], pave the path forward for using better judgement when selecting antibiotic regimes and stimulating the pursuit of novel and improved antibiotics for treating brucellosis. Above all, these findings obligate the need to investigate *Brucella* species’ ecological, genetic and phenotypic mechanisms involved in enabling the pathogen to survive antibiotic treatment.

## 5. Conclusion

*Brucella* populations responsible for human brucellosis are able to develop resistance to RIF and SXT. The emergence of AMR is revealed in specific epidemiological factors, particularly time, geographic location and bacterial species. Findings from this study support the WHO‐recommended main regimen of DOX plus STR but advise cautious stewardship of RIF and SXT to stop further resistance development. Overall, *Brucella* bacteria remain susceptible to DOX, STR, GEN and CIP. Further research into the ecology, genetics and phenotypic mechanisms which underlie resistance may help elucidate how these bacteria evade antibiotic treatment.

## Author Contributions

Gurkan Tut is the sole author of this work and has contributed substantially to all aspects of the study. His contributions include conception and design of the study, data acquisition, formal analysis, methodology, investigation and resources. Gurkan Tut is fully responsible for data curation, validation, visualisation and project administration. Gurkan Tut drafted the manuscript and critically revised the manuscript for important intellectual content.

## Funding

This project in part was supported by resources from BRUCELLA PROJECT XYZ and the U.K. FAO Reference Centre for Antimicrobial Resistance (which receives funding from the Department for Environment, Food & Rural Affairs and U.K. aid funding from the Department of Health and Social Care’s Fleming Fund). The author covered the publication fees for this article.

## Disclosure

The author approved the final version to be published and agrees to be accountable for all aspects of the work. The author alone is responsible for the content and writing of this study.

## Conflicts of Interest

The author declares no conflicts of interest.

## Supporting Information

Additional supporting information can be found online in the Supporting Information section.

## Supporting information


**Supporting Information 1** Figure S1: Screening questions applied to pool relevant studies for data extraction.

**Supporting Information 2** Figure S2: Modified version of the Newcastle–Ottawa Scale for ranking studies on AMR in *Brucella*.

**Supporting Information 3** Figure S3: Interval plot of 1‐sample *Z*‐tests on rifampicin resistance of *Brucella*.

**Supporting Information 4** Figure S4: Interval plot of 1‐sample *Z*‐tests on trimethoprim‐sulfamethoxazole resistance of *Brucella*.

**Supporting Information 5** Figure S5: Funnel plot of the meta‐analysis on the extent of RIF resistance of *Brucella*.

**Supporting Information 6** Figure S6: Funnel plot of the meta‐analysis on the extent of SXT resistance of *Brucella*.

**Supporting Information 7** Table S1: Data items of interest for investigating AMR in *Brucella*.

**Supporting Information 8** Table S2: Tabulated data set of targeted items for investigating AMR in *Brucella*.

**Supporting Information 9** Table S3: MIC_min_ and MIC_max_ averages of *Brucella*.

**Supporting Information 10** Table S4: Meta‐analysis results: summarising MIC_50_ for observational comparisons with CLSI breakpoints and investigating the extent of AMR with meta‐analytical tests.

**Supporting Information 11** Table S5: Summarised meta‐analysis results of GIV WA of MIC_mean_ and its 95% CIs on DOX, RIF, SXT, STR, GEN and CIP for observational comparisons with CLSI breakpoints.

**Supporting Information 12** Table S6: Sensitivity analysed meta‐analysis results on AMR in *Brucella* defined by GIV WA MIC_mean_ in the absence of heterogeneity.

**Supporting Information 13** Table S7: GRADE Summary of Findings. PRISMA_2020_abstract_checklist.docx.

**Supporting Information 14** Table S8: Quality assessment of included studies according to the use of a modified Newcastle–Ottawa Scale.

**Supporting Information 15** The PRISMA 2020 Abstract Checklist includes essential reporting items for abstracts of systematic reviews and for this study can be found in the file ‘*PRISMA_2020_abstract_checklist.docx*’.

**Supporting Information 16** The PRISMA 2020 Checklist provides comprehensive reporting guidelines for systematic reviews, covering aspects such as scope, methodology and findings, and for this study is available in the file ‘*PRISMA_2020_checklist.docx’*.

## Data Availability

The data that support the findings of this study are available in the supporting information of this article.
