# Supplementary material for: Brucella’s Emerging Threat: A Global Systematic Review and Meta‐Analysis Revealing Temporal, Geographic and Species‐Specific Patterns of Antimicrobial Resistance
Source: Vet Med Int. 2026 Feb 10;2026:8689240. doi: 10.1155/vmi/8689240 (PMC12891813; doi:10.1155/vmi/8689240)
Supplement: Supplementary file 1 — Supporting Information 1 Figure S1: Screening questions applied to pool relevant studies for data extraction. [file VMI-2026-8689240-s014.pptx]

## Slide 1
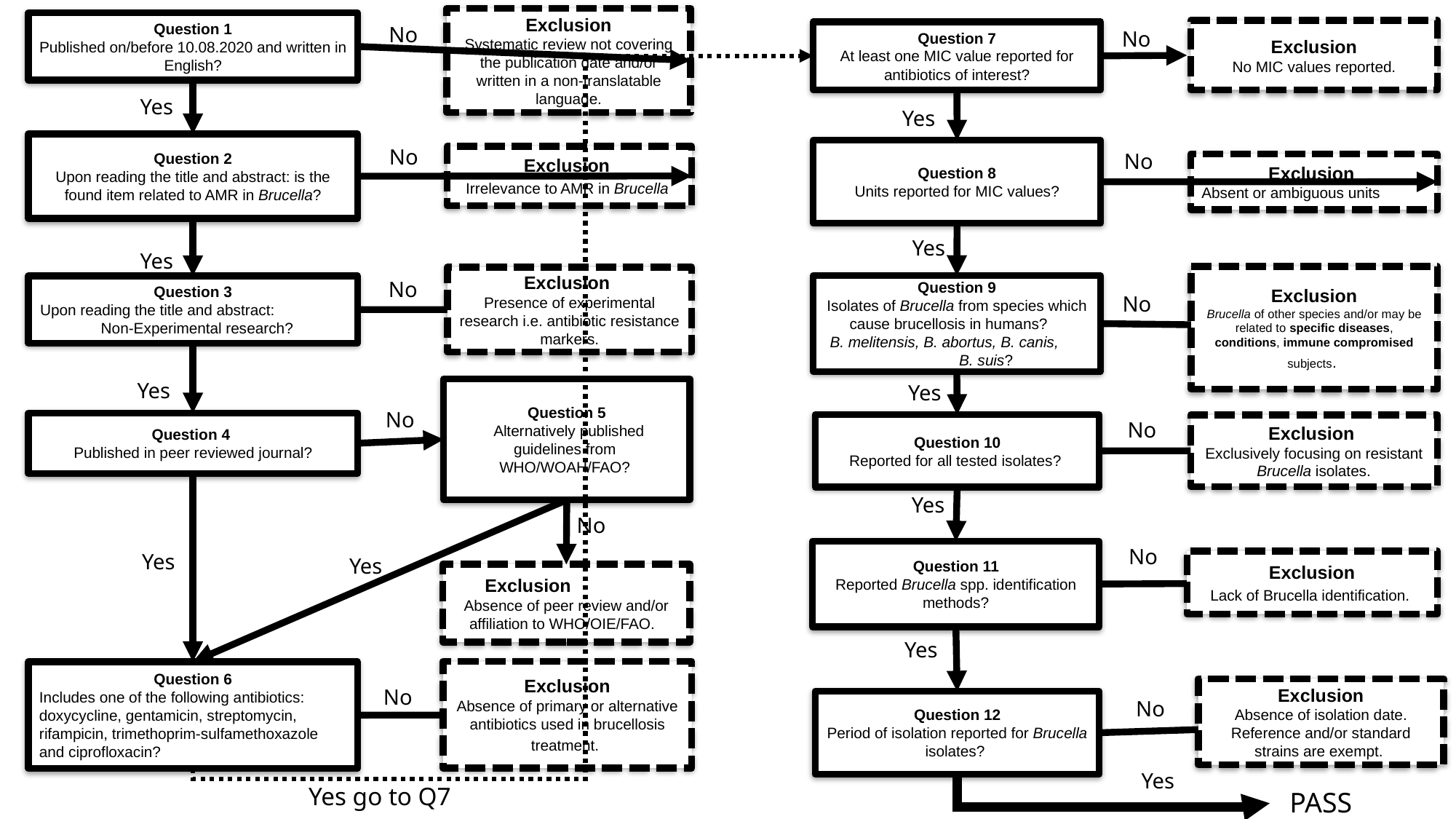

Exclusion
Systematic review not covering the publication date and/or written in a non-translatable language.
Question 1
Published on/before 10.08.2020 and written in English?
No
No
Exclusion
No MIC values reported.
Question 7
At least one MIC value reported for antibiotics of interest?
Yes
Yes
Question 2
Upon reading the title and abstract: is the found item related to AMR in Brucella?
No
Question 8
Units reported for MIC values?
No
Exclusion
Irrelevance to AMR in Brucella
Exclusion
Absent or ambiguous units
Yes
Yes
Exclusion
Brucella of other species and/or may be related to specific diseases, conditions, immune compromised subjects.
Exclusion
Presence of experimental research i.e. antibiotic resistance markers.
No
Question 9
Isolates of Brucella from species which cause brucellosis in humans?
 B. melitensis, B. abortus, B. canis, B. suis?
Question 3
Upon reading the title and abstract: Non-Experimental research?
No
Yes
Yes
Question 5
 Alternatively published guidelines from WHO/WOAH/FAO?
No
No
Question 4
Published in peer reviewed journal?
Question 10
Reported for all tested isolates?
Exclusion
Exclusively focusing on resistant Brucella isolates.
Yes
No
No
Question 11
Reported Brucella spp. identification methods?
Yes
Yes
Exclusion
Lack of Brucella identification.
Exclusion
Absence of peer review and/or affiliation to WHO/OIE/FAO.
Yes
Exclusion
Absence of primary or alternative antibiotics used in brucellosis treatment.
Question 6
Includes one of the following antibiotics: doxycycline, gentamicin, streptomycin, rifampicin, trimethoprim-sulfamethoxazole and ciprofloxacin?
No
Exclusion
Absence of isolation date. Reference and/or standard strains are exempt.
No
Question 12
Period of isolation reported for Brucella isolates?
Yes
Yes go to Q7
PASS
