# Supplementary material for: Brucella’s Emerging Threat: A Global Systematic Review and Meta‐Analysis Revealing Temporal, Geographic and Species‐Specific Patterns of Antimicrobial Resistance
Source: Vet Med Int. 2026 Feb 10;2026:8689240. doi: 10.1155/vmi/8689240 (PMC12891813; doi:10.1155/vmi/8689240)
Supplement: Supplementary file 2 — Supporting Information 2 Figure S2: Modified version of the Newcastle–Ottawa Scale for ranking studies on AMR in Brucella. [file VMI-2026-8689240-s013.doc]

**NEWCASTLE - OTTAWA QUALITY ASSESSMENT SCALE**

**COHORT STUDIES**

Note: A study can be awarded a maximum of one star for each numbered item within the Selection and Outcome categories. A maximum of two stars can be given for Comparability

**Selection**

1) **Origin of *Brucella* isolates** (Representativeness of the exposed cohort)

a) Truly representative isolates of *Brucella* from species which cause brucellosis in humans**🟑**

b) Somewhat representative isolates of *Brucella* from species which cause brucellosis in humans**🟑**

c) Selected group of *Brucella* spp. isolates (e.g. only susceptible or resistant and/or not isolates of *Brucella* from species which cause brucellosis in humans)

d) No description of the derivation of the *Brucella* spp. isolates

2) **Use of reference/standard strains** (Selection of the non-exposed cohort)

a) The standard and/or reference strains of *Brucella* spp. were used as a control during experimentation of the exposed cohort **🟑**

b) The standard and/or reference strains of *Brucella* spp. were used as a control but this was drawn from a different source (i.e. different publications).

c) There is no description of the use or derivation of the standard and/or reference strains of *Brucella* spp. as a control

3) **Document type** (Ascertainment of exposure)

a) Peer reviewed paper from an established journal **🟑**

b) Peer reviewed paper from a non-established journal **🟑**

c) Written report from a company and/or organisation (i.e. WHO/OIE/FAO)

d) No description

4) Demonstration that outcome of interest was not present at start of study

a) yes **🟑**

b) no

**Comparability**

1) **Methods** (Comparability of cohorts on the basis of the design or analysis)

a) Methods described and appropriate to justify findings (i.e. AMR testing methods reported, breakpoints applied, adherence to CLSI standards and guidance and etc.,) **🟑**

b) Adequate control for confounding variables (i.e. the use of multiple AMR testing methods to determine findings, as well as the use of both positive and negative controls, and containing true biological *Brucella* spp. isolates and etc.,) **🟑**

**Outcome**

1) **Interpretation of results** (Assessment of outcome)

a) Independent and blind assessment of results i.e., utilising both statistical methods and CLSI guidance and breakpoints for interpretation **🟑**

b) Only utilising CLSI guidance and breakpoints **🟑**

c) Self report

d) No description

2) **Testing of *Brucella* isolates in accordance with the defined CLSI MIC method** (Was follow-up long enough for outcomes to occur)

a) Yes (appropriate period depends on subject, regimen, testing methods and etc.,)**🟑**

b) No (deviation from the defined CLSI MIC method)

3) **Inclusion of tested *Brucella* isolates: isolate follow up** (Adequacy of follow up of cohorts)

a) Complete follow up - all subjects accounted for **🟑**

b) Subjects lost to follow up unlikely to introduce bias - small number lost - > 5 %, or description provided of those lost **🟑**

c) Follow up rate < 95% and no description of those lost

d) No statement
