# Supplementary figures and images for: Brucella’s Emerging Threat: A Global Systematic Review and Meta‐Analysis Revealing Temporal, Geographic and Species‐Specific Patterns of Antimicrobial Resistance
Source: Vet Med Int. 2026 Feb 10;2026:8689240. doi: 10.1155/vmi/8689240 (PMC12891813; doi:10.1155/vmi/8689240)

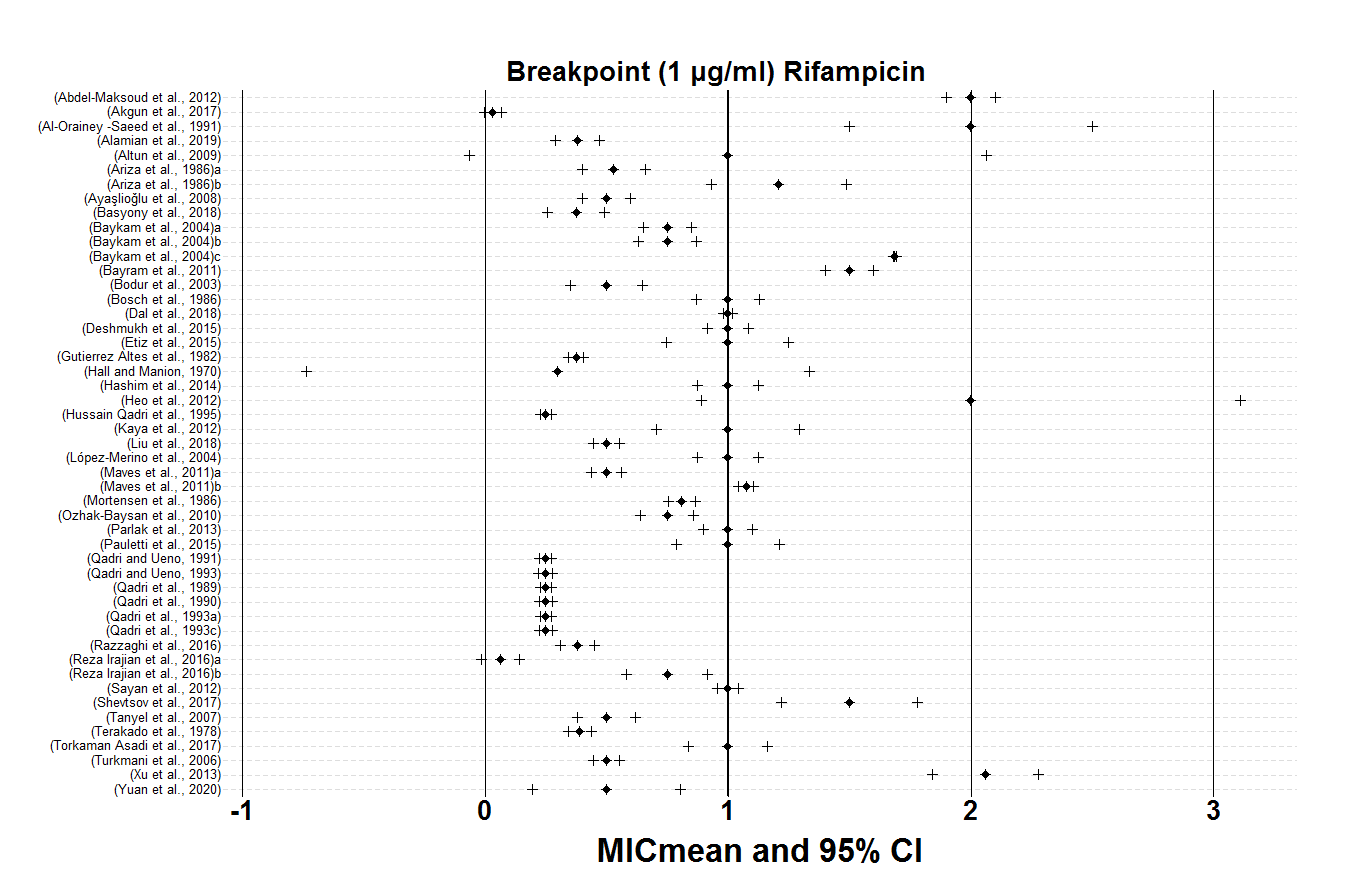

Supplement: Supplementary file 3 — Supporting Information 3 Figure S3: Interval plot of 1‐sample Z‐tests on rifampicin resistance of Brucella. [file VMI-2026-8689240-s012.docx]

**
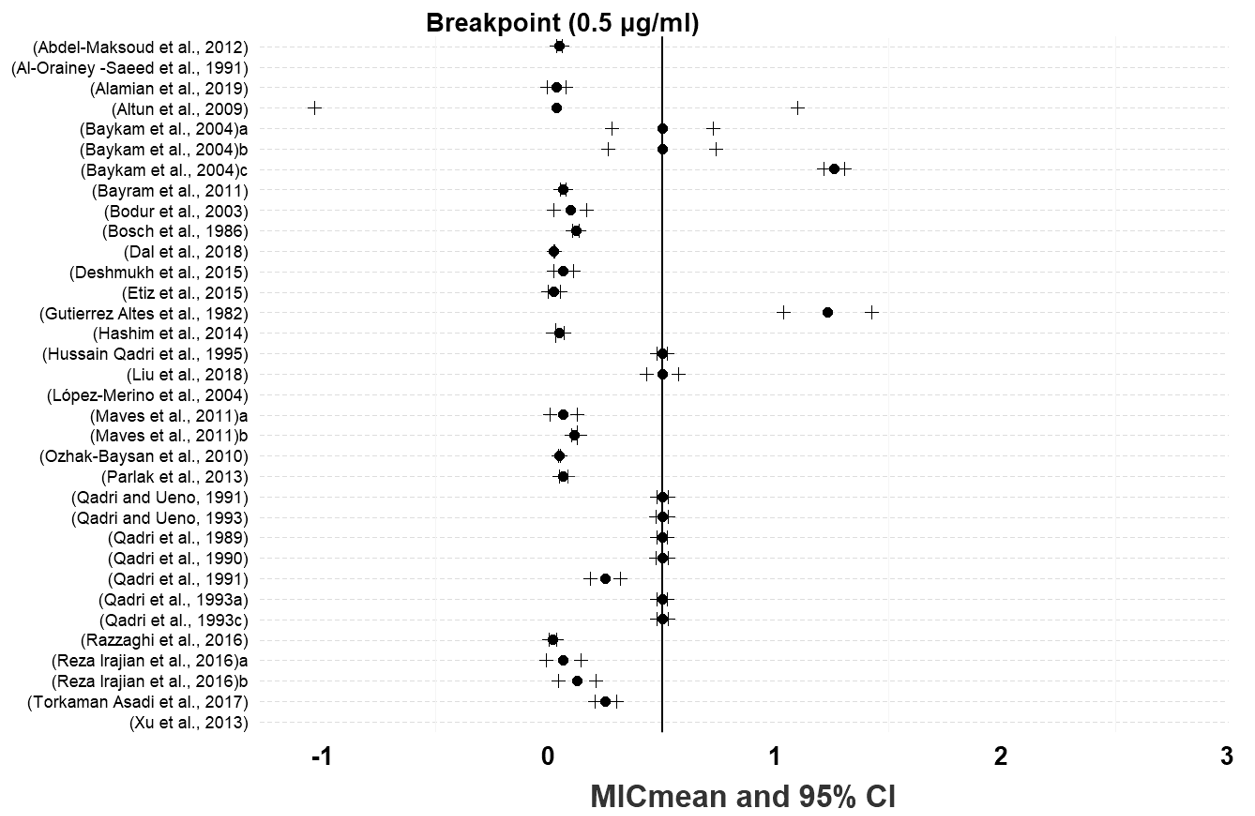
**

Supplement: Supplementary file 4 — Supporting Information 4 Figure S4: Interval plot of 1‐sample Z‐tests on trimethoprim‐sulfamethoxazole resistance of Brucella. [file VMI-2026-8689240-s011.docx]

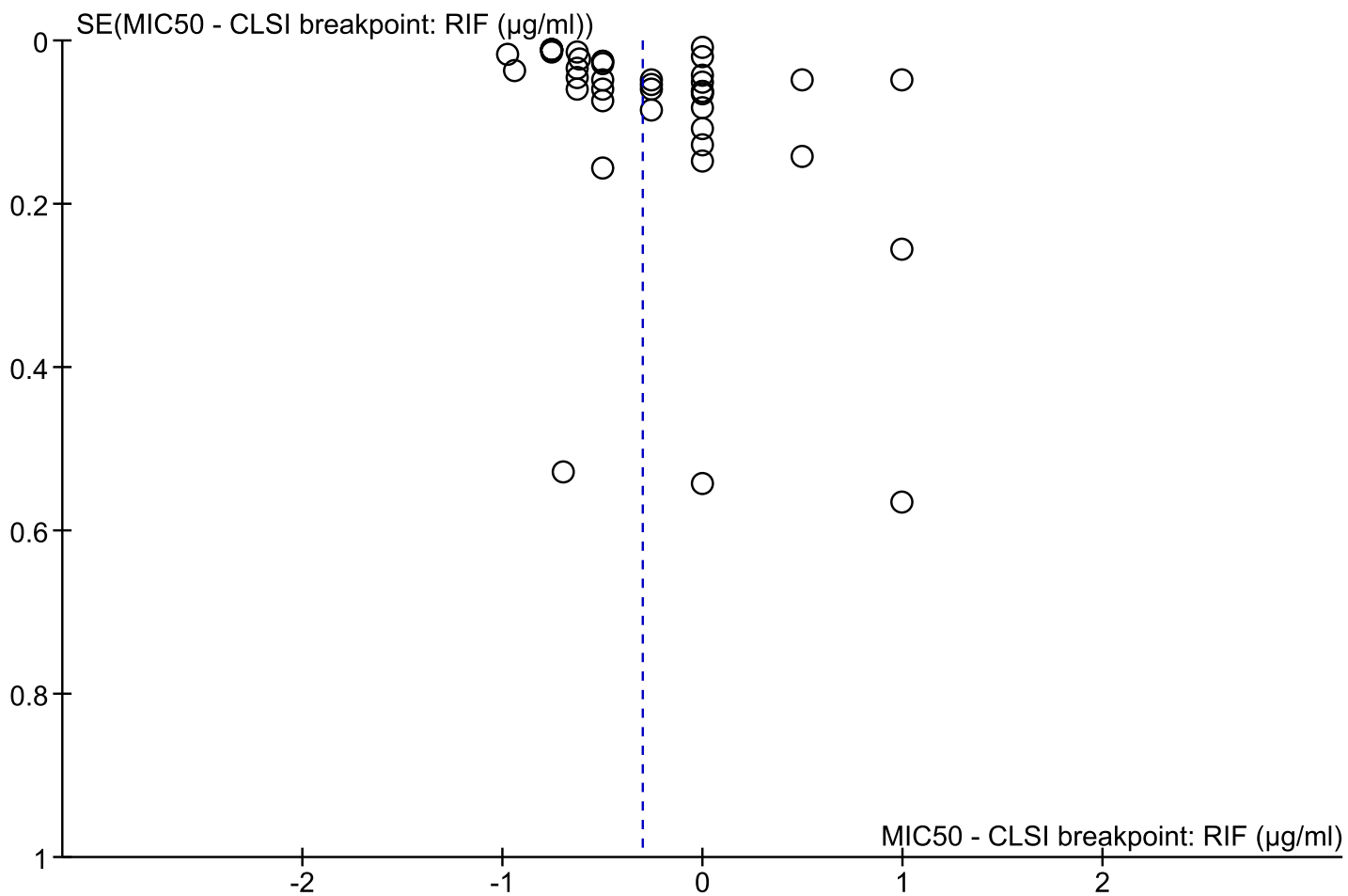

Supplement: Supplementary file 5 — Supporting Information 5 Figure S5: Funnel plot of the meta‐analysis on the extent of RIF resistance of Brucella. [file VMI-2026-8689240-s010.pdf]

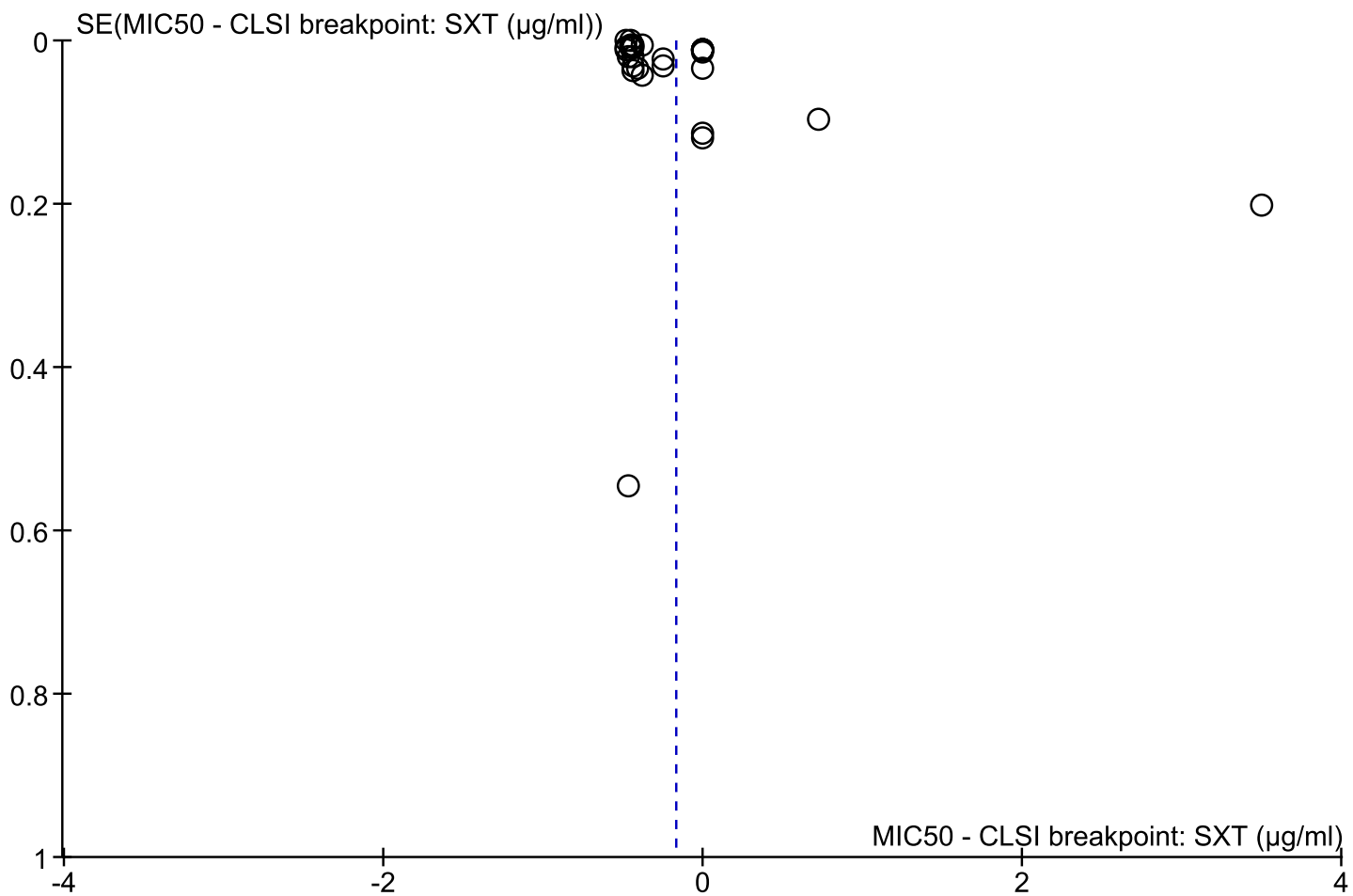

Supplement: Supplementary file 6 — Supporting Information 6 Figure S6: Funnel plot of the meta‐analysis on the extent of SXT resistance of Brucella. [file VMI-2026-8689240-s009.pdf]
