# Supplementary material for: Brucella’s Emerging Threat: A Global Systematic Review and Meta‐Analysis Revealing Temporal, Geographic and Species‐Specific Patterns of Antimicrobial Resistance
Source: Vet Med Int. 2026 Feb 10;2026:8689240. doi: 10.1155/vmi/8689240 (PMC12891813; doi:10.1155/vmi/8689240)
Supplement: Supplementary file 7 — Supporting Information 7 Table S1: Data items of interest for investigating AMR in Brucella. [file VMI-2026-8689240-s008.docx]

| **List** | **Definition** | **Data type** |
| --- | --- | --- |
| **Authors #1** | The writer of the book, article, or document. | Nominal |
| **Resistance #2** | Has the author reported resistance to any antibiotic Yes or No | Ordinal |
| **Antibiotic #3** | The name of the antibiotic | Nominal |
| **Isolates source #4** | Species the isolate has been extracted from. | Nominal |
| **Number of isolates #5** | Number of isolates tested. | Numerical |
| ***Brucella* species #6** | The species of *Brucella* identified. | Nominal |
| ***Brucella* spp. isolation method #6A** | The method used to identify the species of *Brucella* | Nominal |
| **Reference Strain #7** | The name of reference strain used for control purposes | Nominal |
| **Biovar #8** | The Biovar of the *Brucella* species. | Nominal |
| **Source of infection #9** | The source of infection i.e. dead foetuses, herd mixing, unsafe pasture practices etc., | Nominal |
| **Origin of resistance #10** | How is resistance occurring? A gene?, misuse of antibiotics? Evolutionary? Genetic modification? | Nominal |
| **Year #11** | The year the *Brucella* isolates were obtained. | Numerical |
| **Geographic location #12** | The continent of *Brucella* isolate acquisition | Nominal |
| **Country #12 A** | The country of *Brucella* isolate acquisition |  |
| **AMR testing method #13** | The method and/or methods used to test the antibiotic against *Brucella* | Nominal |
| **AMR determination method #13A** | The method used to determine susceptibility and/or resistance | Nominal |
| **AMR interpretation criteria #13B** | Criteria applied to justify susceptibility and/or resistance | Nominal |
| **Standards and guidelines #14** | The standards and guidance followed for producing the results i.e. CLSI and UKAS | Nominal |
| **Breakpoint type #15** | Have the authors employed the use of a break point value: Yes or No, if Yes enter the break point type i.e. clinical, wildtype or ECOFF. | Ordinal & Nominal |
| **Breakpoint value #15A** | The numerical value of the breakpoint in universal units | Numerical |
| **Gene #16** | The reported gene which contributes to resistance | Nominal |
| **MIC #17** | Minimum inhibitory concentration | Numerical |
| **MIC_min_ #18** | Lowest minimum inhibitory concentration | Numerical |
| **MIC_max_ #19** | Highest minimum inhibitory concentration | Numerical |
| **MIC_range_ #20** | The difference between the MIC_min_ and MIC_max_ | Numerical |
| **MIC_50_ #21** | Minimum inhibitory concentration required to inhibit the growth of 50% of *Brucella* isolates. | Numerical |
| **MIC_90_ #22** | Minimum inhibitory concentration required to inhibit the growth of 90% of *Brucella* isolates | Numerical |
| **Financial support #23** | Who supported the study financially | Nominal |
