# Supplementary material for: Brucella’s Emerging Threat: A Global Systematic Review and Meta‐Analysis Revealing Temporal, Geographic and Species‐Specific Patterns of Antimicrobial Resistance
Source: Vet Med Int. 2026 Feb 10;2026:8689240. doi: 10.1155/vmi/8689240 (PMC12891813; doi:10.1155/vmi/8689240)
Supplement: Supplementary file 9 — Supporting Information 9 Table S3: MICmin and MICmax averages of Brucella. [file VMI-2026-8689240-s001.docx]

| **Antibiotic** | **MeanMIC_min_** | SE | SD | **MeanMIC_max_** | SE | SD | **CLSI breakpoint (µg/ml)** |
| --- | --- | --- | --- | --- | --- | --- | --- |
| **DOX** | **0.23** | 0.19 | 1.24 | **1.5** | 0.85 | 5.46 | **≤4** |
| **STR** | **0.42** | 0.08 | 0.54 | **17.34** | 9.55 | 58.11 | **≤8** |
| **RIF** | **0.25** | 0.04 | 0.30 | **3.89** | 0.89 | 6.48 | **≤1** |
| **SXT** | **0.30** | 0.14 | 0.90 | **3.47** | 0.50 | 7.68 | **≤0.5** |
| **GEN** | **0.17** | 0.03 | 0.20 | **8.79** | 7.27 | 43.05 | **≤4** |
| **CIP** | **0.12** | 0.01 | 0.12 | **2.09** | 0.81 | 5.32 | **≤1** |
