# Supplementary material for: Brucella’s Emerging Threat: A Global Systematic Review and Meta‐Analysis Revealing Temporal, Geographic and Species‐Specific Patterns of Antimicrobial Resistance
Source: Vet Med Int. 2026 Feb 10;2026:8689240. doi: 10.1155/vmi/8689240 (PMC12891813; doi:10.1155/vmi/8689240)
Supplement: Supplementary file 11 — Supporting Information 11 Table S5: Summarised meta‐analysis results of GIV WA of MICmean and its 95% CIs on DOX, RIF, SXT, STR, GEN and CIP for observational comparisons with CLSI breakpoints. [file VMI-2026-8689240-s016.docx]

| **Antibiotic** | **Factor** | **DF** | **Mic_mean_** | **95% CI**  **Lower** | **95% CI**  **Upper** |
| --- | --- | --- | --- | --- | --- |
|  |  |  |  |  |  |
| **DOX** | **Overall AMR** | 36 | 0.27 | (0.13 | 0.41) |
|  | **Epidemiological characters** |  |  |  |  |
|  | Human Isolates | 29 | 0.29 | (0.12 | 0.45) |
|  | Mixed Isolates | 6 | 0.21 | (0.14 | 0.27) |
|  |  |  |  |  |  |
|  | *B. melitensis* | 25 | 0.33 | (0.15 | 0.50) |
|  | *Brucella* spp., | 10 | 0.15 | (0.11 | 0.20) |
|  |  |  |  |  |  |
|  | Asia | 24 | 0.11 | (0.09 | 0.13) |
|  | Non-Asian | 11 | 0.21 | (-0.06 | 0.49) |
|  | **Comparison of geography** | 1 | - | - | - |
|  | Isolation year ≤ 2000 | 6 | 0.12 | (0.09 | 0.15) |
|  | Isolation year > 2000 | 24 | 0.33 | (0.15 | 0.51) |
|  | Overlapping isolation years ≤2000 and ≥ 2000 | 4 | 0.22 | (0.12 | 0.33) |
|  |  |  |  |  |  |
|  | **Methodology** |  |  |  |  |
|  | E-test | 22 | 0.34 | (0.16 | 0.53) |
|  | Agar Dilution | 8 | 0.18 | (0.14 | 0.22) |
|  | Broth dilution | 4 | 0.11 | (0.02 | 0.20) |
|  |  |  |  |  |  |
|  | **Meta-biases** |  |  |  |  |
|  | N of isolates ≥ 30 | 26 | 0.12 | (0.10 | 0.14) |
|  | N of isolates < 30 | 9 | 0.78 | (0.46 | 1.10) |
|  |  |  |  |  |  |
|  | **GIV FEM** | 36 | 0.13 | (0.13 | 0.13) |
|  |  |  |  |  |  |
| **RIF** | **Overall AMR** | 48 | 0.78 | (0.56 | 1.01) |
|  | **Epidemiological characters** |  |  |  |  |
|  | Human isolates | 40 | 0.81 | (0.56 | 1.05) |
|  | Mixed isolates | 7 | 0.58 | (0.37 | 0.78) |
|  |  |  |  |  |  |
|  | B. melitensis | 35 | 0.74 | (0.62 | 0.87) |
|  | *Brucella* spp., | 12 | 0.87 | (0.41 | 1.32) |
|  |  |  |  |  |  |
|  | Asia | 36 | 0.76 | (0.49 | 1.03) |
|  | Non-Asian | 11 | 0.87 | (0.61 | 1.13) |
|  |  |  |  |  |  |
|  | Isolation year ≤ 2000 | 14 | 0.44 | (0.37 | 0.51) |
|  | Isolation year 2001-2009 | 14 | 0.97 | (0.71 | 1.22) |
|  | Isolation year ≥ 2010 | 11 | 0.66 | (0.44 | 0.88) |
|  | Overlapping isolation years ≤2000 and ≥ 2000 | 6 | 1.10 | (0.50 | 1.71) |
|  |  |  |  |  |  |
|  | **Methodology** |  |  |  |  |
|  | E-test | 26 | 0.89 | (0.64 | 1.14) |
|  | Agar dilution | 9 | 0.56 | (0.56 | 0.79) |
|  | Broth dilution | 11 | 0.41 | (0.32 | 0.49) |
|  |  |  |  |  |  |
|  | **Meta-biases** |  |  |  |  |
|  | N of isolates ≥ 30 | 39 | 0.71 | (0.60 | 0.82) |
|  | N of isolates < 30 | 8 | 1 | (0.61 | 1.38) |
|  |  |  |  |  |  |
|  | **GIV FEM** | 48 | 1.19 | (1.19 | 1.20) |
| **SXT** | **Overall AMR** | 38 | 0.47 | (0.41 | 0.52) |
|  | **Epidemiological characters** |  |  |  |  |
|  | Human Isolates | 33 | 0.44 | (0.38 | 0.49) |
|  | Mixed Isolates | 4 | 1.06 | (0.50 | 1.63) |
|  |  |  |  |  |  |
|  | B. melitensis | 28 | 0.47 | (0.39 | 0.54) |
|  | *Brucella* spp. | 9 | 0.78 | (0.49 | 1.07) |
|  |  |  |  |  |  |
|  | Asia | 29 | 0.41 | (0.36 | 0.47) |
|  | Non-Asia | 8 | 0.74 | (0.55 | 0.93) |
|  |  |  |  |  |  |
|  | Isolation year ≤2000 | 11 | 0.87 | (0.71 | 1.03) |
|  | Isolation year > 2000 | 22 | 0.27 | (0.23 | 0.32) |
|  | Overlapping isolation years ≤2000 and ≥ 2000 | 3 | 0.16 | (0.03 | 0.29) |
|  |  |  |  |  |  |
|  | **Methodology** |  |  |  |  |
|  | E-test | 22 | 0.19 | (0.15 | 0.22) |
|  | Agar dilution | 7 | 1.71 | (1.44 | 1.98) |
|  | Broth dilution | 7 | 0.69 | (0.56 | 0.83) |
|  |  |  |  |  |  |
|  | **Meta-biases** |  |  |  |  |
|  | N of isolates ≥ 30 | 32 | 0.34 | (0.29 | 0.39) |
|  | N of isolates < 30 | 5 | 3.05 | (2.46 | 3.64) |
|  |  |  |  |  |  |
|  | **GIV FEM** | 38 | 0.03 | (0.03 | 0.04) |
| **CIP** | **Overall AMR** | 40 | 0.30 | (0.26 | 0.34) |
| **GEN** | **Overall AMR** | 31 | 0.43 | (0.35 | 0.52) |
| **STR** | **Overall AMR** | 34 | 1.01 | (0.88 | 1.13) |

**DOX**, doxycycline; **RIF**, rifampicin, **SXT**, trimethoprim-sulfamethoxazole, **CIP**, ciprofloxacin, **GEN**, gentamicin, **STR**, streptomycin. **CI** = confidence interval, **DF** = degrees of freedom.
