# Supplementary material for: Brucella’s Emerging Threat: A Global Systematic Review and Meta‐Analysis Revealing Temporal, Geographic and Species‐Specific Patterns of Antimicrobial Resistance
Source: Vet Med Int. 2026 Feb 10;2026:8689240. doi: 10.1155/vmi/8689240 (PMC12891813; doi:10.1155/vmi/8689240)
Supplement: Supplementary file 12 — Supporting Information 12 Table S6: Sensitivity analysed meta‐analysis results on AMR in Brucella defined by GIV WA MICmean in the absence of heterogeneity. [file VMI-2026-8689240-s002.docx]

| **Antibiotic** | **DF** | **Mic_mean_** | **95% CI**  **Lower** | **95% CI**  **Upper** | **Heterogeneity Test** | |
| --- | --- | --- | --- | --- | --- | --- |
|  |  |  |  |  | I^2^ (%) | P value |
| **DOX** | 5 | 0.13 | (0.12 | 0.13) | 0 | 1.00 |
| **RIF** | 12 | 1 | (0.98 | 1.01) | 0 | 0.56 |
| **SXT** | 11 | 0.50 | (0.49 | 0.51) | 0 | 0.99 |
| **CIP** | 13 | 0.25 | (0.25 | 0.25) | 0 | 1.00 |
| **GEN** | 10 | 0.25 | (0.25 | 0.25) | 0 | 1.00 |
| **STR** | 7 | 0.50 | (0.48 | 0.52) | 0 | 1.00 |
