# Supplementary material for: Brucella’s Emerging Threat: A Global Systematic Review and Meta‐Analysis Revealing Temporal, Geographic and Species‐Specific Patterns of Antimicrobial Resistance
Source: Vet Med Int. 2026 Feb 10;2026:8689240. doi: 10.1155/vmi/8689240 (PMC12891813; doi:10.1155/vmi/8689240)
Supplement: Supplementary file 13 — Supporting Information 13 Table S7: GRADE Summary of Findings. PRISMA_2020_abstract_checklist.docx. [file VMI-2026-8689240-s003.docx]

| **Author and Year^A^** | **Risk of bias^B^** | **Risk of Inconsistency^C^** | **Risk of Indirectness^D^** | **Risk of Imprecision^E^** | **Risk of publication bias^F^** | **Overall**  **GRADE^G^** | **GRADE Rec^H^** |
| --- | --- | --- | --- | --- | --- | --- | --- |
| (Hall and Manion, 1970) | Low | Low | Low | Moderate | Strongly suspected | Low-Moderate | Weak |
| (Terakado et al., 1978) | Low | Low | Low | Moderate | Undetected | Moderate | Strong |
| (Gutierrez Altes et al., 1982) | Low | Low | Low | Moderate | Undetected | Moderate | Strong |
| (Ariza et al., 1986) | Moderate | Low | Low | Moderate | Undetected | Low-Moderate | Weak |
| (Zimmerman et al., 1990) | Low | Low | Low | Moderate | Strongly suspected | Low-Moderate | Weak |
| (Al-Orainey -Saeed et al., 1991) | Low | Low | Low | Moderate | Undetected | Moderate | Strong |
| (Rubinstein et al., 1991) | Low | Low | Low | Moderate | Undetected | Moderate | Strong |
| (Qadri et al., 1993a) | Low | Low | Low | Low | Undetected | Moderate | Strong |
| (Garcia-Rodriguez et al., 1995) | Low | Low | Low | Low | Undetected | Moderate | Strong |
| (Mateu-de-Antonio and Martín, 1995) | Low | Low | Moderate | High | Strongly suspected | Low | Weak |
| (Baykam et al., 2004) | Low | Low | Low | Moderate | Undetected | Moderate | Strong |
| (López-Merino et al., 2004) | Moderate | Low | Low | Low | Strongly suspected | Low-Moderate | Weak |
| (Yamazhan et al., 2005) | Low | Low | Low | Moderate | Undetected | Moderate | Strong |
| (Turkmani et al., 2006) | Low | Low | Low | Moderate | Undetected | Moderate | Strong |
| (Marianelli et al., 2007) | Low | Low | Low | Moderate | Undetected | Moderate | Strong |
| (Tanyel et al., 2007) | Low | Low | Low | Moderate | Undetected | Moderate | Strong |
| (Ayaşlioǧlu et al., 2008) | Low | Low | Low | Moderate | Undetected | Moderate | Strong |
| (Altun et al., 2009) | Low | Low | Low | Low | Undetected | Moderate | Strong |
| (Ozhak-Baysan et al., 2010) | Low | Low | Low | Moderate | Undetected | Moderate | Strong |
| (Bayram et al., 2011) | Low | Low | Low | Moderate | Undetected | Moderate | Strong |
| (Maves et al., 2011) | Low | Low | Low | Moderate | Undetected | Moderate | Strong |
| (Abdel-Maksoud et al., 2012) | Low | Low | Very low | Very Low | Undetected | Moderate-High | Strong |
| (Heo et al., 2012) | Low | Low | Low | Moderate | Undetected | Moderate | Strong |
| (Kaya et al., 2012) | Moderate | Low | Low | Moderate | Undetected | Moderate | Strong |
| (Sayan et al., 2012) | Low | Low | Moderate | Low | Undetected | Moderate | Strong |
| (Parlak et al., 2013) | Low | Low | Low | Moderate | Undetected | Moderate | Strong |
| (Xu et al., 2013) | Low | Low | Low | Moderate | Undetected | Moderate | Strong |
| (Cooke and Perrett, 2014) | Low | Low | Low | Moderate | Undetected | Moderate | Strong |
| (Hashim et al., 2014) | Low | Low | Low | Moderate | Undetected | Moderate | Strong |
| (Deshmukh et al., 2015) | Low | Low | Low | Very Low | Undetected | Moderate | Strong |
| (Etiz et al., 2015) | Low | Low | Low | Moderate | Undetected | Moderate | Strong |
| (Pauletti et al., 2015) | Low | Low | Low | Low | Undetected | Moderate | Strong |
| (Morales-Estrada et al., 2016) | Moderate | Moderate | Low | High | Undetected | Low | Weak |
| (Razzaghi et al., 2016) | Low | Low | Low | Moderate | Undetected | Moderate | Strong |
| (Reza Irajian et al., 2016) | Low | Very low | Very low | Moderate | Undetected | Moderate-High | Strong |
| (Paul et al., 2017) | Moderate | Low | Moderate | Moderate | Undetected | Low | Weak |
| (Shevtsov et al., 2017) | Moderate | Low | Low | Very low | Undetected | Moderate | Strong |
| (Torkaman Asadi et al., 2017) | Low | Low | Moderate | Moderate | Undetected | Moderate | Strong |
| (Basyony et al., 2018) | Low | Low | Low | Moderate | Undetected | Moderate | Strong |
| (Dal et al., 2018) | Low | Low | Low | Moderate | Undetected | Moderate | Strong |
| (Johansen et al., 2018) | Low | Low | Very low | Moderate | Undetected | Moderate | Strong |
| (Liu et al., 2018) | Low | Low | Very low | Moderate | Undetected | Moderate | Strong |
| (Alamian et al., 2019) | Low | Low | Low | Moderate | Undetected | Moderate | Strong |
| (Yuan et al., 2020) | Very Low | Low | Low | Moderate | Undetected | Moderate | Strong |
| (Al-sibai and Qadri, 1990) | Uncertain | High | Moderate | High | Undetected | Low | Weak |
| (Kilic et al., 2008) | Low | Low | Low | Moderate | Undetected | Moderate | Strong |
| (Hussain Qadri et al., 1995) | Moderate | Low | Low | Low | Undetected | Moderate | Strong |
| (Bodur et al., 2003) | Low | Low | Low | Moderate | Undetected | Moderate | Strong |
| (Köse et al., 2005) | Low | Low | Low | High | Undetected | Low | Weak |
| (Qadri and Ueno, 1991) | Moderate | Low | Low | Low | Strongly suspected | Low | Weak |
| (Qadri et al., 1990) | Moderate | Low | Low | Low | Undetected | Moderate | Strong |
| (Al Dahouk et al., 2005) | Low | Moderate | Moderate | High | Undetected | Low | Weak |
| (Bosch et al., 1986) | Moderate | Low | Moderate | Moderate | Undetected | Low | Weak |
| (Qadri and Ueno, 1993) | Moderate | Low | Low | Moderate | Strongly suspected | Low | Weak |
| (Qadri et al., 1991) | Low | Low | Moderate | Moderate | Undetected | Moderate | Strong |
| (Mortensen et al., 1986) | Moderate | Low | Low | Moderate | Undetected | Moderate | Strong |
| (Qadri et al., 1993b) | Low | Low | Low | Low | Undetected | Moderate | Strong |
| (Qadri et al., 1993c) | Low | Low | Low | Low | Undetected | Moderate | Strong |
| (Akgun et al., 2017) | Low | Low | Low | Moderate | Undetected | Moderate | Strong |
| (Qadri et al., 1989) | Uncertain | Low | Low | Low | Strongly suspected | Moderate | Strong |
| (Ransmeier et al., 1951) | Uncertain | Moderate | Moderate | High | Strongly suspected | Low | Weak |

**Table footnotes and explanations for awarding a GRADE**

**A = Author and year of publication** (Harvard style)

**B** = **Risk of bias**: study design and implementation limitations and/or shortcomings. For instance does the study lack: Loss of follow up of subjects? Is selective reporting bias present? Are there other factors limiting the study design and implementation (early termination of experiments and cross over designs). The following grades can be awarded for the risk of bias: **high**, **moderate, uncertain,** or **low**.

**C = Risk of inconsistency** is judged by many factors including differences in estimates of effect, where extensive differences of the same effect exist consistency is questionable. Search for basis of heterogeneity i.e. differences in populations, interventions and outcomes. Clarified heterogeneity is understandable, however unexplained heterogeneity reduces the confidence in results which translate into lower quality of evidence. Other methods for identifying inconsistency includes variation in size effects, and non-overlapping confidence intervals. The lack of overlap in confidence intervals may suggest mean variation among study results are probable. For precisely determining inconsistency the use of statistical tests showing a significance of heterogeneity (P < 0.05) and calculating *I^2^* are appropriate paths. Justifiable downgrading of a study in relation to inconsistency is possible with (1) wide variation in effect, (2) lack of overlap in confidence intervals for the same intervention, (3) statistically significant findings of heterogeneity (P < 0.05) and a (4) large *I^2^* value. Risk of inconsistency can be awarded four grades **very low**, **low**, **moderate,** and **high**.

**D =** **Risk of indirectness**: the investigated research question and the application of PICO (population, interventions, comparisons, and outcomes). Simply put confidence in the results relative to PICO. This can be broken into several important questions investigating the direct evidence, does the study: (1) carry out research in the population and/or systems which we are trying to supply solutions for? (2) Perform research with interventions of interest and compare these interventions with suitable alternatives? (3) Measured outcomes of interest? (4) are the findings generalizable, transferable and externally validated? (5) Does the research evidence satisfy the research question? (6) And finally is the evidence indirect for the population? Risk of indirectness can be awarded the following Grades: **very low**, **low**, **moderate** and **high**.

**E = Risk of imprecision**: are the results precise enough? If not when are they? The following considerations play a key role in decision making (1) sample size (the larger the *n* the more precise the finding), (2) number of events (the higher the number of events the better), (3) confidence intervals, wide intervals bring uncertainty about impact of the effects, while narrow intervals point towards a precise effect. Risk of imprecision is awarded one of the following four grades **very low**, **low**, **moderate** and **high**.

**F = Risk of publication bias**: examining the likelihood of publication bias is critical for determination of over or underestimate of effects because of selective publication of studies. Larger number of subjects leads to a smaller likelihood of publication bias, and therefore if findings are based on small populations this possess a higher risk. Furthermore, if the study is funded by the industry there is a higher likelihood of outcomes benefiting the sponsor, and consequently this poses a high risk of publication bias. The following are the Grades awarded for risk of publication bias: **undetected** (no change in quality), **strongly suspected**, or **very strongly suspected**.

**G** = All criteria (A – F) used to determine the quality of evidence: **High**: ⊕⊕⊕⊕, **Moderate**: ⊕⊕⊕, **Low**: ⊕⊕, **Very Low**: ⊕

Formula: B + C + D + E +F / 5 = mean – 1 and then 4 – (mean -1) = G (produces rank of 1, 2, 3 and 4 (1 being High and 4 being very low)

**H = GRADE recommendation**: reviewing all the evidence (A – G) for the study, and then a GRADE recommendation of favor (**strong**) or against (**weak**) is awarded for the intervention and/or findings. Strong recommendations indicate the majority of people are confident with the intervention and/or findings. Weak recommendations indicate a high possibility of variation in the decision of the intervention and/or findings.
