# Supplementary material for: Brucella’s Emerging Threat: A Global Systematic Review and Meta‐Analysis Revealing Temporal, Geographic and Species‐Specific Patterns of Antimicrobial Resistance
Source: Vet Med Int. 2026 Feb 10;2026:8689240. doi: 10.1155/vmi/8689240 (PMC12891813; doi:10.1155/vmi/8689240)
Supplement: Supplementary file 14 — Supporting Information 14 Table S8: Quality assessment of included studies according to the use of a modified Newcastle–Ottawa Scale. [file VMI-2026-8689240-s004.docx]

| **Study** | **Selection** | | | | **Comparability** | | **Outcome** | | |
| --- | --- | --- | --- | --- | --- | --- | --- | --- | --- |
|  | Origin of *Brucella* isolates | Reference strains | Document type | Outcome of interest | Methods | Confounding variables | Interpretation of results | Outcome  Follow-up | Subject follow-up |
| (Hall and Manion, 1970) | **🟑** | - | **🟑** | **🟑** | **🟑** | - | - | **🟑** | - |
| (Terakado et al., 1978) | **🟑** | **🟑** | **🟑** | **🟑** | **🟑** | - | - | **🟑** | **🟑** |
| (Gutierrez Altes et al., 1982) | **🟑** | **🟑** | **🟑** | **🟑** | **🟑** | - | - | **🟑** | **🟑** |
| (Ariza et al., 1986) | **🟑** | - | **🟑** | **🟑** | **🟑** | - | - | **🟑** | - |
| (Zimmerman et al., 1990) | **🟑** | - | **🟑** | **🟑** | **🟑** | **🟑** | - | **🟑** | **🟑** |
| (Al-Orainey -Saeed et al., 1991) | **🟑** | - | **🟑** | **🟑** | **🟑** | - | - | **🟑** | **🟑** |
| (Rubinstein et al., 1991) | **🟑** | - | **🟑** | **🟑** | **🟑** | - | - | **🟑** | **🟑** |
| (Qadri et al., 1993a) | **🟑** | - | **🟑** | **🟑** | **🟑** | - | - | **🟑** | **🟑** |
| (Garcia-Rodriguez et al., 1995) | **🟑** | **🟑** | **🟑** | **🟑** | **🟑** | - | - | **🟑** | **🟑** |
| (Mateu-de-Antonio and Martín, 1995) | **🟑** | **🟑** | **🟑** | **🟑** | **🟑** | - | - | **🟑** | **🟑** |
| (Baykam et al., 2004) | **🟑** | **🟑** | **🟑** | **🟑** | **🟑** | - | **🟑** | **🟑** | **🟑** |
| (López-Merino et al., 2004) | **🟑** | **🟑** | **🟑** | **🟑** | **🟑** | - | - | **🟑** | **🟑** |
| (Yamazhan et al., 2005) | **🟑** | - | **🟑** | **🟑** | **🟑** | - | - | **🟑** | **🟑** |
| (Turkmani et al., 2006) | **🟑** | **🟑** | **🟑** | **🟑** | **🟑** | - | **🟑** | **🟑** | **🟑** |
| (Marianelli et al., 2007) | **🟑** | **🟑** | **🟑** | **🟑** | **🟑** | - | **🟑** | **🟑** | **🟑** |
| (Tanyel et al., 2007) | **🟑** | - | **🟑** | **🟑** | **🟑** | - | **🟑** | **🟑** | **🟑** |
| (Ayaşlioǧlu et al., 2008) | **🟑** | **🟑** | **🟑** | **🟑** | **🟑** | - | **🟑** | **🟑** | **🟑** |
| (Altun et al., 2009) | **🟑** | - | **🟑** | **🟑** | **🟑** | - | **🟑** | **🟑** | **🟑** |
| (Ozhak-Baysan et al., 2010) | **🟑** | - | **🟑** | **🟑** | **🟑** | - | **🟑** | **🟑** | **🟑** |
| (Bayram et al., 2011) | **🟑** | **🟑** | **🟑** | **🟑** | **🟑** | - | **🟑** | **🟑** | **🟑** |
| (Maves et al., 2011) | **🟑** | **🟑** | **🟑** | **🟑** | **🟑** | - | **🟑** | **🟑** | **🟑** |
| (Abdel-Maksoud et al., 2012) | **🟑** | - | **🟑** | **🟑** | **🟑** | - | **🟑** | **🟑** | **🟑** |
| (Heo et al., 2012) | **🟑** | **🟑** | **🟑** | **🟑** | **🟑** | - | **🟑** | **🟑** | **🟑** |
| (Kaya et al., 2012) | **🟑** | - | **🟑** | **🟑** | **🟑** | - | **🟑** | **🟑** | **🟑** |
| (Sayan et al., 2012) | **🟑** | **🟑** | **🟑** | **🟑** | **🟑** | - | **🟑** | **🟑** | **🟑** |
| (Parlak et al., 2013) | **🟑** | **🟑** | **🟑** | **🟑** | **🟑** | - | **🟑** | **🟑** | **🟑** |
| (Xu et al., 2013) | **🟑** | - | **🟑** | - | **🟑** | - | **🟑** | **🟑** | **🟑** |
| (Cooke and Perrett, 2014) | **🟑** | - | **🟑** | **🟑** | **🟑** | - | **🟑** | **🟑** | **🟑** |
| (Hashim et al., 2014) | **🟑** | **🟑** | **🟑** | **🟑** | **🟑** | - | **🟑** | **🟑** | **🟑** |
| (Deshmukh et al., 2015) | **🟑** | - | **🟑** | **🟑** | **🟑** | - | **🟑** | **🟑** | **🟑** |
| (Etiz et al., 2015) | **🟑** | - | **🟑** | **🟑** | **🟑** | - | **🟑** | **🟑** | **🟑** |
| (Pauletti et al., 2015) | **🟑** | **🟑** | **🟑** | **🟑** | **🟑** | - | **🟑** | **🟑** | **🟑** |
| (Morales-Estrada et al., 2016) | **🟑** | **🟑** | **🟑** | **🟑** | **🟑** | - | **🟑** | **🟑** | - |
| (Razzaghi et al., 2016) | **🟑** | - | **🟑** | **🟑** | **🟑** | - | **🟑** | **🟑** | **🟑** |
| (Reza Irajian et al., 2016) | **🟑** | **🟑** | **🟑** | **🟑** | **🟑** | **🟑** | **🟑** | **🟑** | **🟑** |
| (Paul et al., 2017) | **🟑** | - | **🟑** | **🟑** | **🟑** | - | - | **🟑** | **🟑** |
| (Shevtsov et al., 2017) | **🟑** | **🟑** | **🟑** | **🟑** | **🟑** | - | **🟑** | **🟑** | **🟑** |
| (Torkaman Asadi et al., 2017) | **🟑** | - | **🟑** | **🟑** | **🟑** | - | **🟑** | **🟑** | **🟑** |
| (Basyony et al., 2018) | **🟑** | - | **🟑** | **🟑** | **🟑** | - | **🟑** | **🟑** | **🟑** |
| (Dal et al., 2018) | **🟑** | **🟑** | **🟑** | **🟑** | **🟑** | - | **🟑** | **🟑** | **🟑** |
| (Johansen et al., 2018) | **🟑** | - | **🟑** | **🟑** | **🟑** | **🟑** | **🟑** | **🟑** | **🟑** |
| (Liu et al., 2018) | **🟑** | **🟑** | **🟑** | **🟑** | **🟑** | - | **🟑** | **🟑** | **🟑** |
| (Alamian et al., 2019) | **🟑** | - | **🟑** | **🟑** | **🟑** | **🟑** | **🟑** | **🟑** | - |
| (Yuan et al., 2020) | **🟑** | **🟑** | **🟑** | **🟑** | **🟑** | - | **🟑** | **🟑** | **🟑** |
| (Al-sibai and Qadri, 1990) | - | - | **🟑** | **🟑** | **🟑** | - | - | **🟑** | **🟑** |
| (Kilic et al., 2008) | **🟑** | **🟑** | **🟑** | **🟑** | **🟑** | - | **🟑** | **🟑** | **🟑** |
| (Hussain Qadri et al., 1995) | **🟑** | - | **🟑** | **🟑** | **🟑** | - | - | **🟑** | **🟑** |
| (Bodur et al., 2003) | **🟑** | **🟑** | **🟑** | **🟑** | **🟑** | - | - | **🟑** | **🟑** |
| (Köse et al., 2005) | **🟑** | **🟑** | **🟑** | **🟑** | **🟑** | - | **🟑** | **🟑** | **🟑** |
| (Qadri and Ueno, 1991) | **🟑** | - | **🟑** | **🟑** | **🟑** | - | - | **🟑** | **🟑** |
| (Qadri et al., 1990) | **🟑** | - | **🟑** | **🟑** | **🟑** | - | - | **🟑** | **🟑** |
| (Al Dahouk et al., 2005) | **🟑** | - | **🟑** | **🟑** | **🟑** | - | **🟑** | **🟑** | **🟑** |
| (Bosch et al., 1986) | **🟑** | - | **🟑** | **🟑** | **🟑** | - | - | **🟑** | **🟑** |
| (Qadri and Ueno, 1993) | **🟑** | - | **🟑** | **🟑** | **🟑** | - | - | **🟑** | **🟑** |
| (Qadri et al., 1991) | **🟑** | - | **🟑** | **🟑** | **🟑** | - | **🟑** | **🟑** | **🟑** |
| (Mortensen et al., 1986) | **🟑** | - | **🟑** | **🟑** | **🟑** | - | **🟑** | **🟑** | - |
| (Qadri et al., 1993b) | **🟑** | - | **🟑** | **🟑** | **🟑** | - | **🟑** | **🟑** | **🟑** |
| (Qadri et al., 1993c) | **🟑** | - | **🟑** | **🟑** | **🟑** | - | **🟑** | **🟑** | **🟑** |
| (Akgun et al., 2017) | **🟑** | - | **🟑** | **🟑** | **🟑** | - | **🟑** | **🟑** | **🟑** |
| (Qadri et al., 1989) | **🟑** | - | **🟑** | **🟑** | **🟑** | - | - | **🟑** | **🟑** |
| (Ransmeier et al., 1951) | **🟑** | - | **🟑** | **🟑** | - | - | - | **🟑** | **🟑** |
